# A review of the equine suspensory ligament: Injury prone yet understudied

**DOI:** 10.1111/evj.14447

**Published:** 2024-11-27

**Authors:** Deborah J. Guest, Helen L. Birch, Chavaunne T. Thorpe

**Affiliations:** ^1^ Department of Clinical Science and Services Royal Veterinary College London UK; ^2^ Department of Orthopaedics and Musculoskeletal Science University College London London UK; ^3^ Department of Comparative Biomedical Sciences Royal Veterinary College London UK

**Keywords:** equine, function, injury, structure, Suspensory ligament

## Abstract

The suspensory ligament (SL) is a key component of the elaborate and highly adapted suspensory apparatus in the horse. In addition to contributing to stabilisation of the metacarpophalangeal joint, the SL has a spring like function to reduce the energetic cost of locomotion. Although the SL is highly prone to injury in horses of all ages and competing in a wide range of disciplines, knowledge regarding fundamental structure–function relationships in the SL is lacking, particularly compared with other injury‐prone tendinous structures such as the superficial digital flexor tendon. In this review, we discuss current knowledge of SL composition, structure and mechanical properties and describe the epidemiology, aetiology and pathophysiology of injuries. We evaluate different diagnostic approaches and treatment modalities and identify key areas for future research.

## 1 SUSPENSORY LIGAMENT COMPOSITION AND STRUCTURE

**FIGURE 1:**
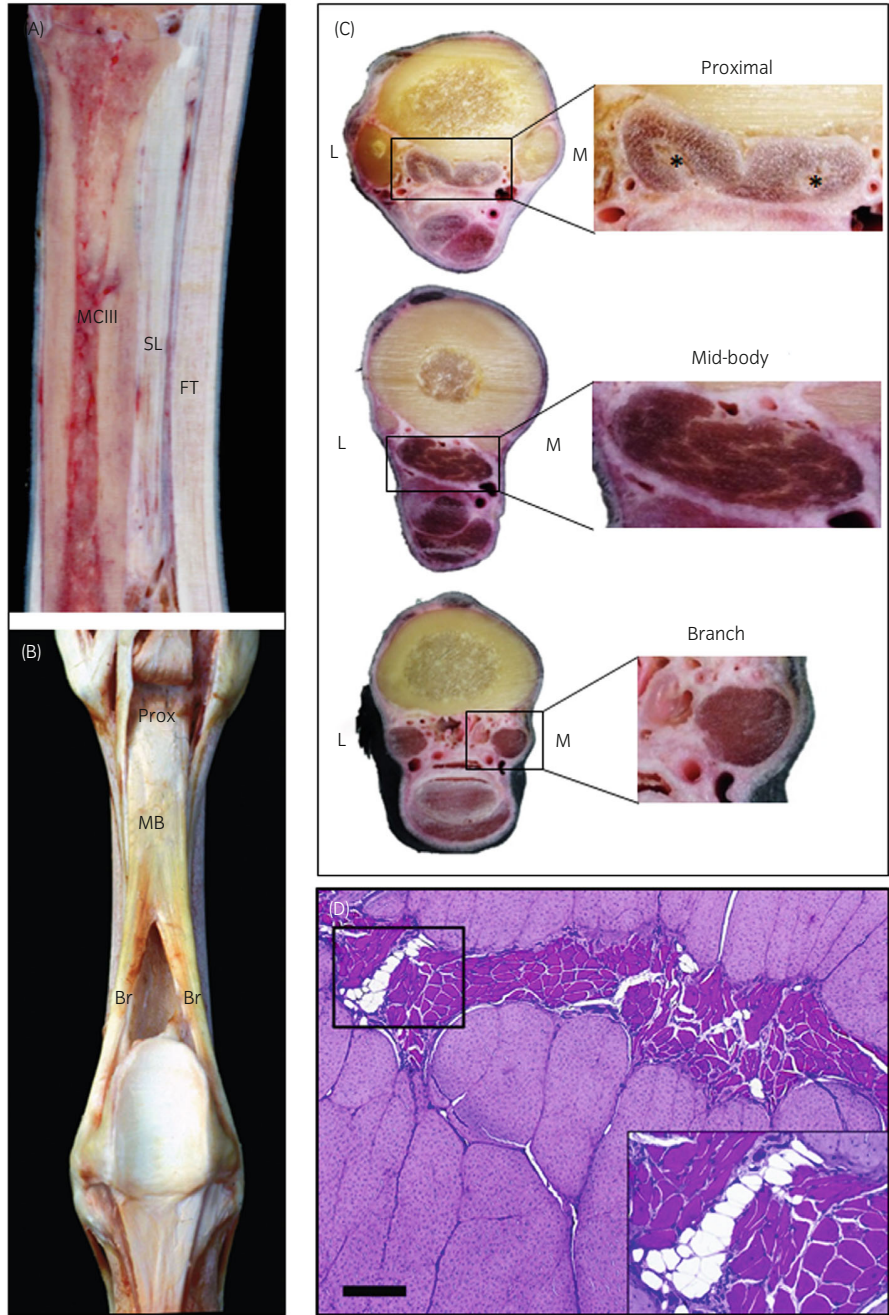
The location, macroscopic and histological appearance of the suspensory ligament. Sagittal section through the forelimb showing the location of the suspensory ligament (SL), third metacarpal bone (MCIII) and flexor tendons (FT) (A). Palmar view showing proximal (Prox), mid‐body (MB) and branch (Br) regions of the suspensory ligament (B). Transverse sections through the forelimb showing the appearance of the proximal, mid‐body and branches of the suspensory ligament. Interfascicular spaces are denoted by * (C). Histological section from the mid‐body showing the presence of muscle fibres within the interfascicular spaces present along the length of the ligament. Scale bar: 200 μm (D). Adapted from Denoix,^1^ Schramme et al.^2^ and Royal Veterinary College Equine Distal Limb Resource (www.rvc.ac.uk/static/review/equine‐distal‐limb/index.html) with permission from the publishers.

The specialised function of the suspensory ligament (SL) is achieved by the highly adapted composition and structural arrangement of the tissue comprising the ligament. The SL originates on the proximo‐palmar/plantar aspect of the third metacarpus/tarsus and continues distally, bifurcating and inserting onto the abaxial surface of the proximal sesamoid bones (Figure 1). The extensor branches extend medially and laterally around the metacarpophalangeal joint, fusing with the common digital extensor tendon.^3^, ^4^ The SL is often divided into three regions, the proximal portion, the body, and the branches (Figure 1B,C). The SL is the anatomical equivalent of the interosseus muscle in other species, and may be referred to by that name in the horse. However, the equine SL has become highly adapted during evolution, with an almost complete loss of muscle fibres except in the proximal region, and an increase in collagen content.^4^ These evolutionary adaptations have resulted in the development of a unique structure, with characteristics reminiscent of tendon, ligament and muscle.^5^

The SL has several functions during locomotion. It is a key component of suspensory apparatus, preventing hyperextension of the metacarpophalangeal joint during stance phase.^3^, ^4^ Indeed, at maximal metacarpophalangeal joint extension the SL and accessory ligaments of the superficial and deep digital flexor tendons are predicted to contribute over half of the total support moment around the joint.^6^ The SL also functions to store and return elastic energy in a similar manner to the forelimb superficial digital flexor tendon (SDFT), reducing the energetic cost of locomotion.^7^ At rest, the SL contributes to the passive stay apparatus, allowing horses to remain standing for long periods of time with little muscular effort.^8^, ^9^

The predominant extracellular matrix (ECM) protein that comprises the SL is type I collagen, with values varying from 34% to 65% dry weight (Table 1).^5^, ^11^ The collagen content varies along the length of the ligament, with a greater collagen content distally, as the proportion of muscular tissue diminishes.^5^ These values are lower than those in the SDFT which is ~75% collagen by dry weight (Table 1). Collagen molecules are stabilised by intermolecular crosslinks, including hydroxylysyl pyridinoline (HP) and lysyl pyridinoline (LP). While HP concentrations are significantly lower in the SL compared with the SDFT, the concentrations of other crosslinks are similar and do not change along the length of the SL, suggesting a broadly similar crosslink profile between the two tissues.^5^, ^12^ Another crosslink, pentosidine, accumulates spontaneously with ageing and therefore can be used as a marker of tissue turnover.^13^, ^14^ Higher pentosidine levels have been reported in the proximal region of the SL compared with the mid‐body and branches, indicating lower turnover of collagen proximally, which may contribute to the increased risk of injury to this region.^5^ Pentosidine levels in the mid‐metacarpal region are higher in the SL than in the SDFT, indicating that the SL is turned over more slowly (Table 1).

**TABLE 1:** Comparison of SDFT and SL composition in the mid‐metacarpal region, measured using standard biochemical techniques in our laboratory.

	Superficial digital flexor tendon	Suspensory ligament
Water (%)	64.91 ± 1.64	67.76 ± 1.30***
Collagen (%)	75.81 ± 8.20	65.11 ± 9.16**
GAG (μg/mg)	10.40 ± 4.86	13.30 ± 5.32*
DNA (μg/mg)	0.54 ± 0.11	0.69 ± 0.17***
Pentosidine (mM/M collagen)	13.83 ± 9.56	17.33 ± 11.65***
HP (mM/M collagen)	737.84 ± 255.58	598.67 ± 231.82*
LP (mM/M collagen)	101.51 ± 108.10	100.23 ± 96.90

*Note*: For further details of methodology see Thorpe.^10^ Significant differences between tissue types are indicated by *: *p < 0.05; **p < 0.01; ***p < 0.001. Data are shown as mean ± SD. *N* = 32.

**FIGURE 2:**
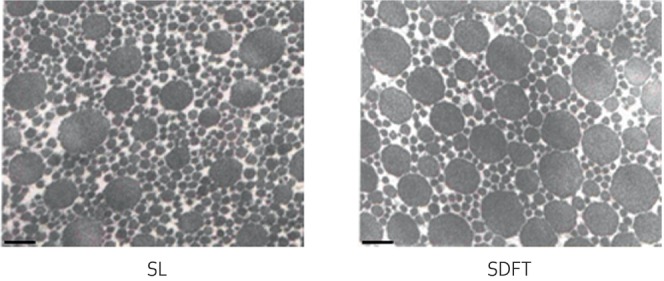
Electron micrographs showing fibril diameters in the SL and SDFT. Scale bar represents 200 nm. For further details of methodology see Smith.^16^

As in other tendons and ligaments, the collagen in the SL is highly aligned and arranged in a hierarchical manner, with type I collagen fibres grouped into fascicles, which are surrounded by interfascicular matrix (also referred to as endotenon).^5^ The diameter of the collagen fibrils follows a bimodal distribution, similar to that in the SDFT.^15^ The mass average fibril diameter, however, is significantly lower in the SL (122 ± 14 nm, *n* = 6) than in the SDFT (169 ± 19 nm, *n* = 6) (Figure 2), although both structures have a higher proportion of small diameter fibrils than the DDFT and CDET. This likely reflects the similar function of the SDFT and SL, as it has been shown that a lower mass average fibril diameter is associated with a lower elastic modulus.^12^

The noncollagenous protein content of the SL is not well established; however, it has been shown that glycosaminoglycan (GAG) content, a measure of proteoglycan levels, varies along the SL length, with the lowest content in the proximal region which increases distally.^5^ The SL also has a higher total sulfated GAG content compared with the SDFT (Table 1). Proteoglycans regulate collagen fibrillogenesis,^17^ but the abundance, location and function of specific noncollagenous proteins within the SL remains to be determined. Indeed, while advanced techniques are now available to provide a proteomic profile of ECM‐rich tissues,^18^ these experiments are yet to be performed for the SL, meaning that our knowledge of proteins that may have an important role in tissue function are yet to be identified.

Unlike other ligamentous structures, the SL contains a high proportion of muscle fibres and other noncollagenous tissue, which varies not only along the length of the ligament but also between fore and hindlimb tendons and in different breeds. The muscle fibres are contained within two interfascicular spaces, one medially and one laterally (Figure 1C, indicated by * in inset). These spaces are evident throughout the majority of the fore and hindlimb SL, and are present slightly distal from the origin, terminating in the middle portion of the ligament branches. They are filled with variable proportions of skeletal muscle fibres, loose connective tissue, adipose tissue as well as blood vessels and nerves.^2^ The sizes of the interfascicular spaces are also heterogeneous, increasing in the mid‐portion and occupying up to 60% of the cross‐sectional area, with the lateral space being significantly larger and containing more muscle fibres than the medial space.^5^, ^19^

Several studies have investigated differences in the proportion of muscle fibres in the SL with age, breed, sex and region; however, there is little agreement in the literature. Initial studies reported no change in muscle content with ageing,^20^ and other studies have not reported horse age.^5^ However, a more comprehensive study found a significant decrease in muscle content, as well as adipose tissue with ageing, with a concomitant increase in connective tissue content, although it should be noted that this study was conducted in standardbreds and so findings may not be applicable to other breeds.^21^ In young Thoroughbreds, high intensity training resulted in a significantly higher collagen content in the SL compared with horses exercised at low intensity.^22^ Indeed, breed specific differences in SL muscle content have also been reported, with 40% greater muscle area in standardbreds compared with Thoroughbreds, and while female standardbreds have a greater SL muscle content than males, there are no sex differences in Thoroughbreds.^20^, ^21^ Further, standardbred hindlimb muscle area content within the SL is greater than in the forelimb; however, this is not the case in the Thoroughbred.^20^

Differences in muscle fibre orientation in the SL have also been reported, with parallel muscle fibres in the proximal forelimb SL which become angled more distally, whereas those in the hindlimb remain parallel throughout the ligament.^5^, ^19^ By contrast, another study reported that muscle fibres in the SL inserted onto their neighbouring collagen fibres at an acute angle; however, it is not clear whether this was throughout the ligament or confined to a specific region.^23^ The functional consequences of differences in muscle content and orientation are yet to be determined but may be related to injury predisposition. Indeed, the muscle fibre types found in the SL are predominantly type I slow twitch fibres which have a short fibre length and high pennation angle, suggesting the ability to generate a large amount of force while producing little work.^23^ This implies that the muscle fibres actively contribute to stabilisation of the forelimb during locomotion, and therefore the high variability seen may affect SL function.

The interfascicular spaces of the SL also house vascular and neural components, with blood supplied proximally from the medial and lateral palmar/plantar arteries, and palmar/plantar metacarpal/tarsal arteries. The blood to the distal SL is also supplied by the lateral and medial palmar/plantar digital arteries.^24^ The SL has an extensive microvascular network throughout its length, with multiple arterioles and venules present not only in the interfascicular spaces but also within the bundles of collagenous fascicles.^24^ This is in contrast to the SDFT, which has a relatively avascular zone that is prone to injury,^25^ suggesting that lack of blood supply does not contribute to increased risk of injury to a particular region of the SL.

The innervation of the SL is relatively well characterised, largely due to work undertaken to understand the nerve supply for diagnostic and treatment purposes, discussed later in this review. The forelimb SL receives its innervation from the deep branch of the lateral palmar nerve (DBLPN), which contains fibres from both the ulnar and median nerve.^26^, ^27^ Similarly, in the hindlimb, the SL is innervated by the deep branch of the lateral plantar nerve, which is a branch of the tibial nerve.^28^ There can be anatomical variations in the DBLPN, with the number of ramifications entering the SL ranging from 2 to 6, and the relationship between the DBLPN and surrounding structures, including the deep plantar arch and accessory ligament of the deep digital flexor tendon (DDFT) also varies.^29^ While this is unlikely to have implications for normal function, it does have important consequences for denervation‐based treatments for SL injury, as discussed below. Nerve fibres are found throughout the SL, particularly in the interfascicular spaces in which the presence of neurovascular bundles have been described.^19^

Little attempt has been made to characterise cell populations within the SL, and even cell nomenclature is not well defined, with cells referred to as fibroblasts, ligamentocytes or desmocytes.^30^, ^31^, ^32^ Studies have measured the DNA content of the SL, which gives a measure of cell number, and varies along the length of the ligament, increasing from proximal to distal.^5^ SL DNA content is also greater than in the SDFT and DDFT,^33^ (Table 1) suggesting a more cellular structure. In addition, there is a very similar gene expression profile between the SL and SDFT, likely reflecting their similar roles as energy stores. However, there are some differences between structures, with lower expression of fibromodulin, MMP‐3 and ‐10 in the SDFT compared with the SL.^33^ There were no significant differences in gene expression with ageing in the SL (Table 2).

**TABLE 2:** Gene expression profile of SL and SDFT.

	Superficial digital flexor tendon	Suspensory ligament
Col1A2	34.93 ± 40.90	29.58 ± 27.21
Col3A1	18.56 ± 22.23	19.06 ± 24.95
Col5A1	1.49 ± 2.26	0.69 ± 0.85
Col12A1	9.51 ± 7.81	15.52 ± 24.32
Aggrecan	5.55 ± 10.24	3.95 ± 4.53
Biglycan	63.27 ± 77.84	75.65 ± 170.55
Decorin	1343.42 ± 1713.18	763.11 ± 429.75
Fibromodulin	32.80 ± 37.79	13.28 ± 12.39*
Lumican	48.50 ± 42.14	58.21 ± 47.97
MMP‐1	0.03 ± 0.06	0.01 ± 0.06
MMP‐3	3.52 ± 4.70	0.70 ± 1.58**
MMP‐9	0.43 ± 1.13	0.84 ± 1.70
MMP‐10	17.46 ± 25.34	2.23 ± 4.36***
MMP‐13	0.16 ± 0.68	0.03 ± 0.06
MMP‐23	0.03 ± 0.06	0.03 ± 0.06
TIMP‐3	55.08 ± 52.55	48.69 ± 53.51
TIMP‐4	0.53 ± 0.85	0.64 ± 0.74
ADAM‐12	0.05 ± 0.06	0.03 ± 0.06
ADAM‐17	1.21 ± 1.24	0.90 ± 1.02
ADAMTS‐2	1.22 ± 1.53	0.63 ± 0.85
Tenascin	1.86 ± 2.77	1.15 ± 1.70
Scleraxis	3.79 ± 5.60	2.51 ± 3.00
COMP	2674.33 ± 3255.43	2111.21 ± 1898.04

*Note*: For further details of methodology see Thorpe.^10^ Significant differences between tissue types are indicated by *: *p < 0.05; **p < 0.01; ***p < 0.001. Data are shown as mean ± SD. *n* = 32.

While some studies have investigated cell behaviour in vitro, the focus of this research has been to develop treatments rather than understand fundamental cell biology.^30^, ^34^, ^35^, ^36^ With recent single cell sequencing studies in tendon unveiling the complex cellular heterogeneity within these tissues,^37^, ^38^, ^39^ it is important that future studies identify, characterise and localise individual cell populations within the SL to understand the role of each population in ligament health, ageing and disease.

## 2 SUSPENSORY LIGAMENT MECHANICAL PROPERTIES

The mechanical properties of the SL have also been investigated, although these remain less well defined than those of the distal limb tendons. Mechanical testing of the entire SL revealed large differences in regional strain patterns under loading, with greater strains in the branches compared with the mid‐body and proximal regions. There were also differences in strain response within the mid‐body, with the distal region experiencing higher strains than the proximal region.^40^ This is supported by studies that have mechanically tested small sections of the SL, and shown that strain is significantly greater in the branches of the ligament than in the mid‐body, while elastic modulus and stiffness are significantly lower.^5^

**FIGURE 3:**
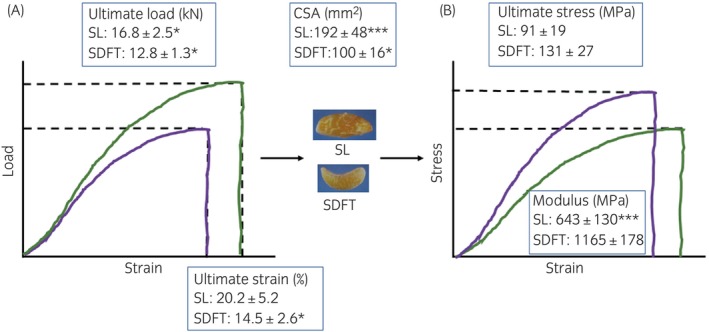
Stylised force/elongation curve (A) and stress–strain curve (B) for SL (green) and SDFT (purple) overlayed with data obtained in vitro from experimental measurement of mechanical properties. Data are shown as mean ± SD (*n* = 6). Significant differences between structures are indicated by *: *p < 0.05; **p < 0.01; ***p < 0.001.

Mechanical testing of the body of the SL suggests a more compliant tissue than the SDFT. Data from our laboratory (unpublished) shows that the elastic modulus is significantly lower than the SDFT (Figure 3). The ultimate strength of the SL is higher than the SDFT, as expected for a larger structure; however, the ultimate stress is significantly lower (Figure 3).

The forces and strains experienced by the SL in vivo have also been determined by implanting transducers within the ligament. Strains of ~3.5% were measured in the walk, increasing to ~5.5% in the trot. There was also an effect of ground surface, with greater strains on pavement than sand.^41^ SL strains are also affected by shoeing, with increased strains when heel wedges or egg‐bar shoes were applied compared with a neutral shoe.^42^ In support of in vitro data, transducer‐measured strains within the SL branches are higher than in the body, reaching ~5.4% at the walk and ~9.1% at the trot.^43^ However, transducer implantation results in alterations in SL mechanical properties in the days following implantation, reducing stress and strain measured post‐mortem.^43^ Further, as transducer implantation is highly invasive, this technique is no longer considered an acceptable method for measuring tendon and ligament strains.

More recently, noninvasive modelling approaches have identified that the forces and strains experienced by the SL peak during midstance, with maximum force and strain exceeding 11 kN and 20% respectively during trotting.^44^, ^45^, ^46^ These strains are higher than those measured in vivo, which may be due to overestimations as a result of the modelling parameters used.^44^ There are also differences in strain between limbs, with higher strains in the forelimb SL, increasing to over 30% in the gallop, whereas strains in the hindlimb remain similar to those in the trot.^7^ Speed within a pace will also likely affect SL loading environment, with greater tarsal flexion and metacarpophalangeal joint extension measured in extended trot compared with a collected trot presumably leading to greater SL strains.^47^ Strains in the SL can also vary according to abrupt changes in ground surface, suggesting that surface inconsistency may contribute to increased risk of injury.^48^

The effect of training on SL morphology and composition has also been studied, with no differences in SL cross‐sectional area between trained and untrained horses.^49^, ^50^ However, collagen content in the SL is increased and GAG content is decreased in high intensity compared with low intensity training.^22^ This may indicate an accelerated loss of muscle tissue within the SL as a result of high intensity exercise. The limited changes in SL properties post‐training suggest that this structure is already highly optimised for efficient function, and little enhancements can be gained through conditioning.

## 3 EPIDEMIOLOGY OF SUSPENSORY LIGAMENT INJURY

The majority of research into the epidemiology of SL injury has been in the Thoroughbred racehorse. In this population, SL injuries are common with an incidence of 2.7 per 1000 horse starts,^51^ and a prevalence of 3.6%–10%.^52^, ^53^, ^54^ Although SL injuries represent a significant proportion of all injuries (11.5%–21.5%), they occur less frequently than bone fractures and injury to the SDFT.^51^, ^55^ In racehorses, 89% of tendon and ligament injuries are to the SDFT and 11% are to the SL.^56^ This may be why, compared with bone fracture and tendon injury, limited research has been performed to identify risk factors for SL injury in racing Thoroughbreds.

Horses with subclinical to mild injury of the SL ligament are more likely to develop a severe injury.^57^ Abnormality of the SL detected during a prerace physical inspection was associated with an increased risk of injury during the race.^58^, ^59^ Further, horses suffering from an SL injury prior to the start of their 2‐year‐old race season have a decreased racing ability.^54^ Given that up to 68% of Thoroughbred horses will not race again after sustaining an SL injury,^60^ a better understanding of the risk factors predisposing to SL injury would be of benefit.

Several studies have demonstrated an association between age and SL injury.^57^, ^61^ Two‐year‐old horses have less SL desmitis than older horses,^60^ the risk of SL injury in 3‐ and 4‐year‐olds is around twice as high than in 2‐year‐olds,^53^ and horses over 5 years old have a five times higher risk of SL injury than 2‐year‐olds.^53^

Other factors associated with risk may include sex, as entire males have been found to be at greater in one study.^53^ Whereas another study found a significant association of SL injury and trainer but not gender.^56^ The race type may influence risk, with increased numbers occurring in chases rather than flat racing and on all‐weather tracks compared with turf.^61^ By contrast, no seasonal association with SL injury has been observed.^51^ An association between mild SL injuries and both the height of shoe toe grabs and distance trained in the preceding week has been found.^57^ Likewise, horses that had undergone a superior accessory ligament desmotomy to treat an SDFT injury were at 5.5 times greater risk of developing SL injury than horses managed nonsurgically.^62^ These findings suggest that changes to load distribution and exercise may influence the risk of an SL injury.

SL injury is also a significant problem in other breeds and horses competing in other disciplines. In standardbred racehorses, SL injury is the most frequent musculoskeletal injury,^63^ whereas in racing Arabians and Thoroughbreds, SDFT injury is more common.^64^ SL injuries are reported in horses used in barrel racing, although they are less common than foot pain and osteoarthritis.^65^ In event horses, SDFT injuries are more common than SL injuries,^66^, ^67^ whereas in general purpose horses, showjumpers and dressage horses the SL is the most commonly injured site.^66^ Interestingly, dressage horses have more hindlimb SL injuries,^68^ whereas it is predominantly the forelimb SL which is affected in all other disciplines.^53^ This may reflect the increased weight bearing undertaken during collection and more advanced dressage movements which result in increased loading on the hindlimbs.^68^

In other breeds and disciplines, there are fewer studies reporting the return to work and re‐injury rates following an SL injury than in racehorses. In one study, only up to 25% of Dutch Warmblood horses and 18% of Standardbreds showed full‐functional recovery.^69^ However, another study found that following an apical fracture, 56%–88% of Standardbreds returned to racing, and this was not affected by the degree of associated SL damage.^70^ In leisure horses with SL branch injuries, 24% returned to the same level of work 2 years after injury, with 41% of horses returning at a lower level of work, and the remainder being unable to return to a ridden career.^71^

Therefore, while it is clear that SL injuries are a major problem across multiple disciplines, very few specific risk factors have been identified.

### 3.1 Aetiology and pathophysiology of suspensory ligament injury

Following injury to the SL relatively little is known about what happens to the SL matrix or cellular components. Histological studies have reported changes in collagen fibre organisation and fibroblast viability.^72^, ^73^ Ultrasonography has also revealed mineralisation in the SL branches, although this is not always associated with lameness.^74^

While most SL injuries occur in the absence of any underlying condition or known genetic variation, there are certain disorders that can directly affect the SL and therefore predispose to injury. Indeed, a significant heritability of 0.05 to 0.17 has been reported for SL injury in Thoroughbreds in Hong Kong,^52^ suggesting a genetic component to injury risk. Furthermore, the SL is affected in some heritable conditions. For example, Quarter horses suffer from an autosomal recessive condition, Hereditary Equine Regional Dermal Asthenia (HERDA). Affected homozygous horses have fragile and hyperextensible skin, which makes the horses unsuitable for riding. However, the ultimate tensile strength of the SDFT, DDFT and SL are also significantly reduced.^75^ An increased risk of SL injury has not been reported in these horses, but this may be due to their inability to undertake ridden work.

Degenerative suspensory ligament desmitis (DSLD) is a chronic, progressive disease that occurs in a range of breeds with an estimated heritability of 0.22.^76^, ^77^ Affected horses usually develop bilateral or quadrilateral lameness and do not improve with rest.^78^ In addition to the SL, other tissues are also affected.^79^, ^80^ The disease is characterised by collagen fibre disorganisation and increased proteoglycan accumulation and is also known as equine systemic proteoglycan accumulation (ESPA).^79^, ^80^ Specifically, increases in aggrecan, aggrecanases, such as ADAMTS4 (A‐disintegrin‐and‐metalloproteinase‐with‐thrombospondin‐like‐motifs 4) and ADAMTS5, and IαI (inter‐alpha‐trypsin‐inhibitor, a marker of chronic inflammation) have been observed in affected SLs.^81^ An abnormal isoform of decorin that has reduced binding to TGF‐β1, has also been found to accumulate in affected tissues. This is associated with increased TGF‐β1 expression that may affect collagen turnover.^80^ Altered TGF‐β‐signalling target genes have also been reported in cells from affected horses.^82^ Increased levels of BMP2 have also been reported in SLs and skin from affected horses.^83^, ^84^ Recent work has suggested that DSLD is a polygenic disease,^85^ but causal variants have yet to be identified. A genome‐wide association study showed enrichment of pathways associated with ECM homeostasis, proteoglycan metabolism and hedgehog signalling.^77^ It is likely that DSLD is a complex disease with additional risk factors, for example, an association between DSLD and pituitary pars intermedia dysfunction (PPID) has been reported.^86^ This may be due to dysregulation of cortisol metabolism.^87^ The link between endocrine disorders and SL injury therefore warrants further investigation.^88^

Injury to the SL has also been associated with injuries to other musculoskeletal tissues. For example, partial transection of the medial branch of the SL increases the strain on the MC3 lateral condylar bone surface in vitro,^89^ suggesting that SL injury may increase the risk of lateral condylar fracture. Indeed, moderate lesions in SL branches are associated with an increased risk of suspensory apparatus failure and metacarpal condylar fracture.^57^, ^90^ Magnetic resonance imaging (MRI) has also revealed that most horses with SL pathology have cortical bone pathology.^68^ Equally, all cases of fatal fracture to the proximal sesamoid bones, third metacarpal or condyles had partial or complete lacerations of the SL,^91^ and at least 70% of horses with splint bone fractures had desmitis of the SL.^92^ Furthermore, sesamoiditis in yearlings leads to a five times greater risk of a SL branch injury when they commence training.^93^ Sesamoiditis is also associated with subclinical ultrasonographical changes in the SL branch.^94^ Damage to the SL can also occur in the presence of an exotosis on the palmar or plantar cortex of MC3 or third metatarsal (MT3) bone.^95^ However, no association was observed between exotoses on the dorsoproximal aspect of MT3 and SL damage.^96^

Therefore, despite the high frequency of SL injuries and their association with other MSK injuries, there is a paucity of studies to identify the causal mechanisms and the changes to the matrix and cellular components that occur following an injury.

### 3.2 Diagnosis of suspensory ligament pathology

Diagnosis of SL pathology is complicated by the inaccessibility of the SL for palpation and lack of localising signs; while acute injuries may be hot with slight swelling and pain on palpation, chronic injuries do not display these signs.^97^ Therefore, SL injury is usually diagnosed by a combination of diagnostic anaesthesia (nerve blocking) and imaging.

When considering the application of nerve blocks, localising pain originating from the SL can be complex due to anatomical variations in nerve supply and the presence of surrounding structures in close proximity, particularly in the proximal region, which may result in inadvertent infiltration of the carpal or tarsal joints and sheaths.^29^, ^98^, ^99^, ^100^ In the hindlimb, anaesthesia of the DBLPN can also abolish pain originating from more distal structures in the limb, including the tarsal joint.^101^ More recent studies have refined diagnostic analgesic techniques, demonstrating that a single small volume injection adjacent to the DBLPN in the hindlimb is sufficient to provide anaesthesia to the SL; however, the risk of affecting surrounding structures remains.^102^, ^103^ In the forelimb, direct synovial communications between the carpometacarpal joint and the proximal SL have recently been identified,^104^ explaining why specific analgesia of the SL remains challenging. These findings highlight the importance of combining diagnostic anaesthesia with imaging for a definitive diagnosis of SL pathology.

**FIGURE 4:**
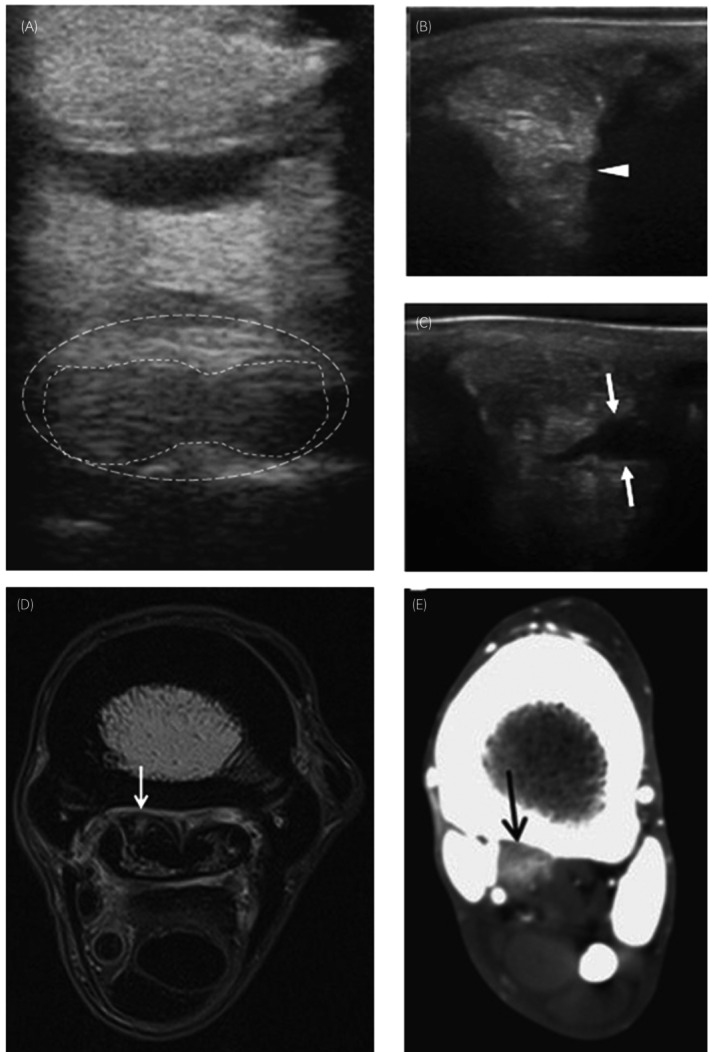
Imaging modalities used to diagnose pathology within the SL. Sagittal ultrasound image of the proximal forelimb SL showing a focal hypoechoic region with fibre disruption (A). Longitudinal ultrasound of the lateral forelimb SL branch performed under weightbearing (B) and nonweightbearing (C). Note the split which is markedly more apparent during nonweightbearing examination. MRI of the proximal forelimb showing a focal injury to the medial lobe of the SL (D). Contrast enhanced CT image of the proximal hindlimb demonstrating enlargement of the SL accompanied by increased contrast (E). Images adapted from Gaschen et al.,^105^ Werpy et al.^106^ and Werpy and Denoix^28^ with permission from the publishers.

Several imaging techniques are used to diagnose SL pathology, with the most common being ultrasound, and more recently MRI. Several abnormalities within the SL are visible on ultrasound, including enlargement, loss of definition of ligament margins, hypoechoic regions (either well or poorly defined) and areas with a diffuse reduction in echogenicity in the proximal SL (Figure 4A).^97^, ^107^ Lesions within SL branches are also detectable ultrasonographically, appearing as anechoic zones, with hypoechoic regions, generally accompanied by heterogeneous echogenicity and disrupted fibrillar pattern.^108^, ^109^ Indeed, ultrasonography of nonlame horses revealed that ~7% of Thoroughbreds used for flat racing, ~30% of National Hunt horses, 58% of showjumping Warmbloods and 20% of working Quarter Horses had evidence of abnormalities in the SL branches.^110^, ^111^, ^112^, ^113^ While only 5% of the showjumpers with SL abnormalities went on to develop clinical signs of SL branch injury within 1 year, more comprehensive studies are required to fully establish if these subclinical abnormalities predispose to injury.

There can be significant inter‐operator variability during ultrasound assessment of the SL, particularly in the proximal region, which should be taken into consideration when diagnosing SL disease.^114^ Ultrasound imaging of the proximal SL is also complicated by the presence of muscle and fat within this region, and therefore an in‐depth knowledge of SL anatomy combined with multiple ultrasound approaches is required for accurate diagnosis.^28^ In addition to imaging from the palmar aspect of the limb, it is recommended that medial and lateral imaging is performed to detect lesions on the edge of the SL, along with examination using the angle contrast technique with the limb slightly flexed and nonweightbearing, enabling visualisation of the origin of the SL and ability to distinguish collagen fibres from muscle and fat.^28^, ^115^ Indeed, this nonweightbearing technique is also successful at visualising longitudinal splits in the SL branches which are not visible when the horse is bearing weight (Figure 4B,C).^106^ This approach has been used to identify an increase in SL abnormalities between 2‐ and 4‐year‐old Quarter horses used for cutting.^116^ Doppler ultrasonography, which provides the ability to detect increases in blood flow, has also been investigated as a diagnostic tool for SL disease, with studies showing good agreement between Doppler and B‐mode ultrasonography when assessing pathology in SL branches.^117^

Another recently developed ultrasound‐based technique is elastography, which is able to estimate tissue stiffness with good reliability and repeatability between operators when imaging normal SLs.^118^ In addition, elastography of pathological SLs showed that acute lesions were softer than chronic lesions, and stiffness increased with healing. Elastography therefore has the potential to improve characterisation and monitoring of SL disease; however, elastography was unable to detect some small, proximal lesions,^119^ limiting its applicability for diagnosis of injuries in this region. Elastography is also yet to be used for clinical diagnosis of SL disease in the horse.

While these ultrasound‐based techniques are successful in identifying a high proportion of SL injuries, MRI provides advances in early diagnosis of SL injuries (Figure 4D),^28^ which is likely key for successful treatment. Development of MRI units able to accommodate a horse limb has provided significant advances in imaging of the SL, with initial studies on disarticulated limbs demonstrating an excellent ability to resolve distal limb structures including the SL.^120^ Studies comparing MRI and ultrasound with histology of the normal SL showed that MRI was able to detect the interfascicular spaces present within the ligament, which could not be resolved using ultrasound.^2^, ^19^ MRI of nonlame horses also demonstrated large variability in appearance of the SL, particularly in the proximal region, which should be taken into account during image interpretation.^121^ Changes detectable on MRI include SL thickening, adhesions between the SL and adjacent bony structures, loss of fibre integrity and the presence of core lesions.^105^, ^122^ MRI is also able to detect biochemical changes in the absence of overt structural changes, leading to earlier diagnosis and potentially improved prognosis^123^; however, it is important that normal variations are not mis‐interpreted as pathology.^124^ MRI is particularly useful for diagnosis of proximal SL disease, with different weightings allowing resolution of structures often difficult to visualise on ultrasound or x‐ray, including the abaxial margins of the SL.^125^ Indeed, direct comparison of ultrasonography and MRI for diagnosis of SL disease revealed that sonography had fair sensitivity but poor specificity in the proximal region of the ligament.^101^ MRI also provides increased sensitivity when diagnosing suspensory branch injuries, with only one quarter to half the lesions visible by MRI detectable ultrasonographically.^126^, ^127^

As well as enabling better diagnosis of lesions within the SL, MRI is able to simultaneously detect pathologies involving other structures, with diagnosticians considering MRI most useful in these cases compared with those only involving the SL.^28^, ^128^ Osseous contusions or sclerosis of the palmar cortex of MC3 at the SL origin can be a feature of SL disease, and the degree of resorption of the palmar cortex correlated with degree of sclerosis and severity of irregularity in the margin of the SL.^123^, ^129^ MRI can also detect adhesions between exostoses on MC3 and the suspensory ligament, which are otherwise only detectable during surgery.^130^ Other studies have identified the presence of bone marrow lesions within MT3 at the SL origin associated with enlargement of the SL and proximal SL pathology.^131^, ^132^ It is interesting that irregularities in the dorsal margin of the SL and sclerosis of the MC3 have also been observed in contralateral, control limbs, which may indicate that these changes are not necessarily indicative of pathology, or conversely that subclinical abnormalities are present in the contralateral limb of horses diagnosed with SL disease in the opposite limb.^133^ Bony abnormalities associated with SL pathology and mineralisation of SL body and branches have also been diagnosed using computed tomography (CT).^134^, ^135^, ^136^ In addition, CT can detect enlargement of the SL itself, and, when combined with injection of contrast agent, can visualise changes in blood flow within the ligament,^28^, ^136^ indicating that CT may be a useful diagnostic tool when MRI facilities are not available (Figure 4E).

Nuclear scintigraphy is another imaging approach that has been used to detect SL pathology, with scintigraphy used in combination with radiography to diagnose avulsions of the origin of the SL.^137^ Scintigraphy has been used with ultrasound to localise lameness when nerve blocks are unsuccessful,^138^ but may not detect SL pathology in all cases.^139^

A large body of work has been undertaken to establish the most effective technologies and protocols for accurate diagnosis of SL pathology. Ultrasound remains the mainstay of diagnosis in the field, while MRI provides increased sensitivity and early diagnosis and so should be considered in the absence of any abnormalities present ultrasonographically. Other imaging techniques such as CT and scintigraphy have also been used to diagnose SL disease, generally alongside other imaging modalities rather than in isolation.

### 3.3 Treatment of suspensory ligament injuries

The standard treatment for an SL injury is box rest and controlled exercise. Modifications to shoeing are also often performed following an SL injury. Toe wedges reduce the strain placed on the SL,^139^ and a wide toe and narrow branches redistribute the pressure from the toe to the heels.^140^ However, these adaptations may increase the strain in other structures in the distal limb.^141^

As re‐injury of the SL following conservative management is common, this has driven the development of other techniques to try to improve tissue healing and result in better outcomes.

#### 3.3.1 Surgical techniques

Surgical interventions to remove damaged tissue have been used for injuries to the SL branches,^142^ but large‐scale studies are lacking. Desmoplasty (surgical splitting of the injured SL) following proximal SL desmitis has been performed for horses with chronic lameness with all horses returning to work,^143^ but long‐term follow‐up was not performed. Ligament splitting has also been combined with microfracture in experimental models of proximal SL injury,^144^ but there are no clinical reports using this approach.

The most common surgical intervention is neurectomy. It has been proposed that proximal SL desmitis of the hindlimbs can lead to compression and damage of the DBLPN.^145^ Neurectomy of DBLPN is effective in many chronically lame horses,^146^ with reports demonstrating that 43%–78% of horses return to their previous, or higher, level of work.^147^, ^148^, ^149^ The variation in success may be related to discipline and other factors such as conformation.^149^ The resolution of lameness may be due to relieving pain in the SL itself, or neuropathic pain caused by compression of the DBLPN by the inflamed SL. Although neurectomy does not lead to changes in the size of the SL,^147^ histological studies have revealed that it does result in muscle atrophy of the proximal SL,^150^, ^151^, ^152^ which may predispose it to re‐injury. Although neurectomy is widely used, there are no reports on the long‐term re‐injury rates following the procedure. As it may cause limb hyposensitivity, neurectomy may also restrict the competition use of the horse depending on the rules of the associated regulatory body.

#### 3.3.2 Extracorporeal shock wave therapy

Extracorporeal shock waves (ESWs) are pulses of high‐energy pressure waves that are used in both human and veterinary orthopaedics. Early studies on the use of ESW for SL injuries demonstrated possible improvements in return to work rates in sport/general use horses, although these studies lacked control groups and compared outcomes to other published clinical data.^153^, ^154^ A comparison of ESW therapy and platelet‐rich plasma (PRP) in the treatment of Western performance horses revealed that horses treated with ESW were more likely (3.8 times) to return to work than horses treated with PRP.^155^ In humans, ESW has been reported to have an immediate pain relieving effect but this was not found to be significant in horses with chronic proximal suspensory desmitis.^156^

Experimental studies using collagenase‐induced SL injuries revealed significant improvements in ultrasound parameters including lesion size, fibre alignment score and echogenicity in ESW‐treated SLs compared with untreated controls.^157^ However, histologically there was only a difference in metachromasia, which appeared more focal in the ESW‐treated SLs, with no differences in cellular appearance or collagen III staining.^157^ ESW therapy also significantly decreased the size of an experimentally induced lesion and resulted in significantly more small collagen fibrils along with increased TGF‐β1 expression.^158^ However, the effect of ESW therapy on the normal, healthy SL revealed that 6 weeks after treatment there was a decrease in GAG and collagen synthesis while overall DNA content remained unchanged.^159^ The tissue also appeared more disorganised with increased MMP14 and collagen I gene expression.^160^

Therefore, despite some promising clinical and experimental results, the effect of ESW therapy on healthy and injured SL matrix and cellular components remains unclear.

#### 3.3.3 High‐power laser and ultrasound‐based therapeutics

High‐power laser is often used in human sports medicine with the belief that it improves healing and reduces pain. In 150 sport horses suffering from tendon and SL injuries laser therapy was found to be safe and treated horses exhibited an overall re‐injury rate of ~20%.^161^ However, without a control group and given the variation in the initial injury type it is not possible to draw conclusions on the efficacy of the treatment.

Using a model of SL branch injury, where lesions were created mechanically in 12 warmblood horses, lesions treated with laser therapy were significantly smaller than control lesions and showed a significantly increased Doppler signal during the treatment indicating more blood flow.^162^ Laser‐treated lesions also showed improved histological scores and lower levels of collagen type III expression.^163^ However, the mechanical properties of the tissue were not assessed.

There is also a single study reporting the use of low‐frequency ultrasound in the treatment of 23 horses with SL injuries.^164^ Eighty‐seven percent of the horses returned to work, but re‐injury rates were not reported and no control group was used. Another ultrasound‐based technique that has been investigated for the treatment of SL pathology is percutaneous ultrasonic debridement; 3 horses that underwent this procedure had improved lameness scores and all returned to work.^165^ However, the small sample number and lack of a control group in this single study means that this technique requires further validation as a therapeutic for SL injury.

#### 3.3.4 Platelet‐rich plasma

Biological therapies to aid tissue regeneration by boosting cell number and/or activity have become increasingly popular over the past 20 years. In addition to their role in blood clotting, platelets release a wide variety of growth factors that may aid tissue regeneration. Increasing platelet concentrations to create PRP is relatively simple and it has therefore been a popular treatment for treating tissue injuries. However, there are few reports that have determined the effect of PRP on SL injuries.

In vitro, PRP affects gene expression in cultured SL cells with an increased ratio of collagen type I to collagen type III, increased cartilage oligomeric matrix protein (COMP) and decreased MMP‐13.^35^ Furthermore, SL tissue explants cultured with PRP have reduced IL‐1β and increased IL1Ra production.^34^ While this is suggestive of beneficial changes, larger‐scale analyses using global gene expression profiling or proteomics have not been reported.

In vivo, PRP injected into lesions in three horses with chronic SL injuries did not result in ultrasonographic improvements, but the horses were able to return to their pre‐injury level of work after 6 months, with no injury recurrence within 20 months.^166^ In contrast, a larger study treated 11 horses with acute suspensory branch injuries with PRP. Although they found all the lesions improved ultrasonographically, only five of the horses returned to their previous level of work.^167^ In racehorses, nine Standardbreds with SL injuries were all able to return to racing following PRP treatment. However, compared with noninjured horses they had significantly reduced earnings per start during the first year after returning to racing and a significant reduction in the number of starts in the third year.^168^ Further, control groups were not used in any of these studies and long‐term re‐injury rates were not recorded.

A later study reported that PRP treatment of yearling Thoroughbreds with sesamoid bone inflammation and SL branch injuries resulted in no significant differences in money earned and races started between horses treated with PRP and control horses treated with saline.^169^ More recently, a larger study on sports horses, involving 22 control horses and 46 horses treated with PRP for chronic hindlimb suspensory desmopathy, found that significantly more horses treated with PRP returned to their previous level of activity compared with the controls.^170^ However, 25 horses in a third group were treated with concentrated bone marrow aspirate and these horses were found to have better lameness scores than the PRP‐treated group at both short‐ and long‐term follow‐up.^170^

#### 3.3.5 Bone marrow and stem cell‐based therapies

Acellular bone marrow aspirate has also been investigated for its therapeutic use. However, these studies have only been performed in vitro. Acellular bone marrow added to cultured ligament fibroblasts was found to increase COMP and total protein synthesis to a greater degree than PRP.^30^ It was also found to stimulate decorin and COMP gene expression by SL explant cultures to a greater degree than PRP.^36^ Nevertheless, it is considerably more invasive to derive than PRP and the effect of using acellular bone marrow alone to treat SL injuries in horses has not been reported.

Whole bone marrow aspirate containing the cellular and acellular fractions was used to treat forelimb SL injuries of 13 Standardbred and 17 Thoroughbred horses. Approximately 70% of both groups returned to racing and had 5 or more starts.^171^ Similarly, a combination of PRP and bone marrow mononuclear cells was used to treat 13 horses with either SL or SDFT injuries and 84.6% returned to work.^172^

These early promising results led to work that focussed on the mesenchymal stromal/stem cells (MSCs) present in the bone marrow and other tissues. Umbilical cord blood (UCB)‐MSCs were used to treat 22 warmblood horses with SL injuries, of which 68% returned to work.^173^ A more recent study treated Thoroughbred racehorses with SL branch injuries with allogeneic UCB‐MSCs, followed by multiple treatments with autologous BMSCs and 71% of horses returned to racing for an average of 29.5 months.^174^ However, none of the aforementioned studies included a control group or assessed re‐injury rates.

MSCs which have been primed towards the tendon lineage (tenogenic primed MSCs) express more type I collagen and less smooth muscle actin than nonprimed MSCs and have better adherence to tendon and ligament tissue explants in vitro.^175^ A case study first reported the safe application of allogeneic tenogenic primed MSCs for an SL injury,^176^ and this was followed by a larger study which treated 68 horses with an SL injury with allogeneic tenogenic primed MSCs in PRP and found 82.4% had returned to the previous level of performance after 2 years and only 17.6% of horses had re‐injured. However, while promising, there was no control group with which to compare the re‐injury rates.^177^ A recent randomised control trial comparing injection of a proprietary formulation of tenogenic primed MSCs with saline injections showed greater improvement in ultrasound and lameness scores in the MSC‐treated SLs up to 112 days post‐treatment. Further, follow‐up after 2 years showed that re‐injury rates were significantly greater in saline treated compared with MSC‐treated horses.^178^ A more recent refinement of MSC‐based therapeutics focuses on microvesicles from MSCs, which are proposed to contain many of the factors that underpin MSC efficacy in aiding tissue repair. Microvesicles from adipose‐derived stem cells were injected into the injured SL of one horse and no adverse events were observed.^179^ However, no conclusions on efficacy can be made.

#### 3.3.6 Gene therapy

Gene therapy approaches have also been trialled as a treatment for SL injuries. Gene therapy involves the injection of expression vectors to express specific genes beneficial for tissue repair. It therefore offers a more targeted approach than the use of PRP or MSCs. The injection of a plasmid expressing vascular endothelial growth factor 164 (VEGF164) and fibroblast growth factor 2 (FGF2) under the control of constitutive promoters was performed in 10 horses with SDFT or SL injury.^180^, ^181^ Eight of the 10 horses were able to return to work with no re‐injury during the 12‐month follow‐up. However, only three of the horses had SL injuries and one of these did not return to work. This study also had no control group and so, while promising, limited conclusions on effectiveness can be drawn.

In summary, while many treatments are being used to treat SL injuries, there is limited data on return to work and re‐injury rates. The lack of control groups, or even robust baseline data, makes it difficult to determine efficacy and further research should address this unmet need.

## 4 CONCLUSIONS

It is evident that the SL in the horse has evolved into a complex and heterogeneous structure, reflecting its crucial role in supporting the distal limb and providing efficient locomotion. Despite these specialisations, the SL is prone to injury and while a variety of treatments are available, the long‐term outcome of these therapeutics remains uncertain. The basic structure of the SL has been characterised, however, studies investigating the relationships between cellular and matrix composition and function, and how these are affected by injury are lacking. This knowledge gap hampers the ability to develop novel therapeutics to effectively treat SL injury.

## FUNDING INFORMATION

The authors received no financial support for the authorship and publication of this article.

## CONFLICT OF INTEREST STATEMENT

The authors declare no conflicts of interest.

## AUTHOR CONTRIBUTIONS

**Deborah J. Guest:** Conceptualization; writing – original draft; writing – review and editing. **Helen L. Birch:** Writing – original draft; writing – review and editing. **Chavaunne T. Thorpe:** Conceptualization; writing – original draft; writing – review and editing.

## DATA INTEGRITY STATEMENT

Not applicable.

## ETHICAL ANIMAL RESEARCH

Not applicable.

## INFORMED CONSENT

Not applicable.

## PEER REVIEW

The peer review history for this article is available at https://www.webofscience.com/api/gateway/wos/peer-review/10.1111/evj.14447.

## Data Availability

Data sharing is not applicable to this article as no new data were created or analysed in this study.

## References

[1] Denoix JM . Essentials of clinical anatomy of the equine locomotor system. 1st ed. Boca Raton: CRC Press; 2019.

[2] Schramme M , Josson A , Linder K . Characterization of the origin and body of the normal equine rear suspensory ligament using ultrasonography, magnetic resonance imaging, and histology. Vet Radiol Ultrasound. 2012;53(3):318–328. 22332890 10.1111/j.1740-8261.2011.01922.x

[3] Budras KD , Sack WO , Rock S . Anatomy of the horse. 3rd ed. Hanover, Germany: Schluetersche; 2001.

[4] Dyce KM , Sack WO , Wensing CJG . Textbook of veterinary anatomy. 3rd ed. Saint Louis, MO: Elsevier; 2002.

[5] Souza MV , Van Weeren PR , Van Schie HT , Van De Lest CH . Regional differences in biochemical, biomechanical and histomorphological characteristics of the equine suspensory ligament. Equine Vet J. 2010;42(7):611–620. 20840576 10.1111/j.2042-3306.2010.0089.x

[6] NaT B , Pandy MG , Kawcak CE , Mcilwraith CW . Force‐ and moment‐generating capacities of muscles in the distal forelimb of the horse. J Anat. 2003;203(1):101–113. 12892409 10.1046/j.1469-7580.2003.00206.x PMC1571149

[7] Biewener AA . Muscle‐tendon stresses and elastic energy storage during locomotion in the horse. Comp Biochem Physiol B Biochem Mol Biol. 1998;120(1):73–87. 9787779 10.1016/s0305-0491(98)00024-8

[8] Gussekloo SWS , Lankester J , Kersten W , Back W . Effect of differences in tendon properties on functionality of the passive stay apparatus in horses. Am J Vet Res. 2011;72(4):474–483. 21453148 10.2460/ajvr.72.4.474

[9] Schuurman S , Kersten W , Weijs W . The equine hind limb is actively stabilized during standing. J Anat. 2003;202:355–362. 12739613 10.1046/j.1469-7580.2003.00166.x PMC1571089

[10] Thorpe CT , Streeter I , Pinchbeck GL , Goodship AE , Clegg PD , Birch HL . Aspartic acid racemization and collagen degradation markers reveal an accumulation of damage in tendon collagen that is enhanced with aging. J Biol Chem. 2010;285(21):15674–15681. 20308077 10.1074/jbc.M109.077503 PMC2871433

[11] Riemersma DJ , De Bruyn P . Variations in cross‐sectional area and composition of equine tendons with regard to their mechanical function. Res Vet Sci. 1986;41(1):7–13. 3764104

[12] Birch HL . Tendon matrix composition and turnover in relation to functional requirements. Int J Exp Pathol. 2007;88(4):241–248. 17696905 10.1111/j.1365-2613.2007.00552.x PMC2517317

[13] Sell DR , Monnier VM . Structure elucidation of a senescence cross‐link from human extracellular matrix. Implication of pentoses in the aging process. J Biol Chem. 1989;264(36):21597–21602. 2513322

[14] Verzijl N , Degroot J , Thorpe SR , Bank RA , Shaw JN , Lyons TJ , et al. Effect of collagen turnover on the accumulation of advanced glycation end products. J Biol Chem. 2000;275(50):39027–39031. 10976109 10.1074/jbc.M006700200

[15] Shikh Alsook MK , Gabriel A , Salouci M , Piret J , Alzamel N , Moula N , et al. Characterization of collagen fibrils after equine suspensory ligament injury: an ultrastructural and biochemical approach. Vet J. 2015;204(1):117–122. 25795168 10.1016/j.tvjl.2015.02.011

[16] Smith T . The relationship between tendon morphology and function. London, UK: University College London; 2006.

[17] Yoon JH , Halper J . Tendon proteoglycans: biochemistry and function. J Musculoskelet Neuronal Interact. 2005;5(1):22–34. 15788868

[18] Peffers MJ , Thorpe CT , Collins JA , Eong R , Wei TK , Screen HR , et al. Proteomic analysis reveals age‐related changes in tendon matrix composition, with age‐ and injury‐specific matrix fragmentation. J Biol Chem. 2014;289(37):25867–25878. 25077967 10.1074/jbc.M114.566554 PMC4162187

[19] Bischofberger AS , Konar M , Ohlerth S , Geyer H , Lang J , Ueltschi G , et al. Magnetic resonance imaging, ultrasonography and histology of the suspensory ligament origin: a comparative study of normal anatomy of warmblood horses. Equine Vet J. 2006;38(6):508–516. 17124840 10.2746/042516406x156109

[20] Wilson DA , Baker GJ , Pijanowski GJ , Boero MJ , Badertscher RR . Composition and morphologic features of the interosseous muscle in standardbreds and Thoroughbreds. Am J Vet Res. 1991;52(1):133–139. 2021241

[21] Shikh Alsook MK , Antoine N , Piret J , Moula N , Busoni V , Denoix J‐M , et al. Morphometric analyses of the body and the branches of the normal third interosseous muscle (suspensory ligament) in standardbreds. Anat Histol Embryol. 2013;42(6):461–470. 23464541 10.1111/ahe.12038

[22] Birch HL , Wilson AM , Goodship AE . Physical activity: does long‐term, high‐intensity exercise in horses result in tendon degeneration? J Appl Physiol. 2008;105(6):1927–1933. 18832761 10.1152/japplphysiol.00717.2007

[23] Soffler C , Hermanson JW . Muscular design in the equine interosseus muscle. J Morphol. 2006;267(6):696–704. 16511864 10.1002/jmor.10433

[24] Williams MR , Arnoczky SP , Pease AP , Stick JA . Microvasculature of the suspensory ligament of the forelimb of horses. Am J Vet Res. 2013;74(12):1481–1486. 24274884 10.2460/ajvr.74.12.1481

[25] Kraus‐Hansen A , Fackelman G , Becker C . Preliminary studies on the vascular anatomy of the equine superficial digital flexor tendon. Equine Vet J. 1992;24:46–51. 1555540 10.1111/j.2042-3306.1992.tb02778.x

[26] Muylle S , Desmet P , Simoens P , Lauwers H , Vlaminck L . Histological study of the innervation of the suspensory ligament of the forelimb of the horse. Vet Rec. 1998;142(22):606–610. 9682420 10.1136/vr.142.22.606

[27] Keg PR , Schamhardt HC , Weeren PRV , Barneveld A . The effect of the high palmar nerve block and the ulnar nerve block on lameness provoked by a collagenase‐induced tendonitis of the lateral branch of the suspensory ligament. Vet Q. 1996;18(sup2):103–105. 8933685

[28] Werpy NM , Denoix JM . Imaging of the equine proximal suspensory ligament. Vet Clin North Am Equine Pract. 2012;28(3):507–525. 23177129 10.1016/j.cveq.2012.08.005

[29] Milner PI , Dimmock O , Barnes K . Neurovascular variations in the proximal plantar metatarsal region of the horse. Equine Vet J. 2022;54(4):782–787. 10.1111/evj.13507 34569652

[30] Smith JJ , Ross MW , Smith RK . Anabolic effects of acellular bone marrow, platelet rich plasma, and serum on equine suspensory ligament fibroblasts in vitro. Vet Comp Orthop Traumatol. 2006;19(1):43–47. 16594543

[31] Dyson S . Is degenerative change within hindlimb suspensory ligaments a prelude to all types of injury? Equine Vet Educ. 2010;22(6):271–274.

[32] Smith RKW . Regenerative medicine in equine orthopaedics: what and when? UK‐Vet Equine. 2020;4(1):8–13.

[33] Thorpe CT . Extracellular matrix synthesis and degradation in functionally distinct tendons. London: Institute of Orthopaedics and Musculoskeletal Science, University College London; 2010.

[34] Bonilla‐Gutiérrez AF , Castillo‐Franz C , López C , Álvarez ME , Giraldo CE , Carmona JU . Equine suspensory ligament and tendon explants cultured with platelet‐rich gel supernatants release different anti‐inflammatory and anabolic mediators. Biomed Pharmacother. 2018;108:476–485. 30241051 10.1016/j.biopha.2018.09.065

[35] McCarrel T , Fortier L . Temporal growth factor release from platelet‐rich plasma, trehalose lyophilized platelets, and bone marrow aspirate and their effect on tendon and ligament gene expression. J Orthop Res. 2009;27(8):1033–1042. 19170097 10.1002/jor.20853

[36] Schnabel LV , Mohammed HO , Jacobson MS , Fortier LA . Effects of platelet rich plasma and acellular bone marrow on gene expression patterns and DNA content of equine suspensory ligament explant cultures. Equine Vet J. 2008;40(3):260–265. 18267879 10.2746/042516408X278030

[37] Zamboulis DE , Marr N , Lenzi L , Birch HL , Screen HRC , Clegg PD , et al. The interfascicular matrix of energy storing tendons houses heterogenous cell populations disproportionately affected by aging. Aging Dis. 2024;15(1):295–310. 37307816 10.14336/AD.2023.0425-1 PMC10796100

[38] Kendal AR , Layton T , Al‐Mossawi H , Appleton L , Dakin S , Brown R , et al. Multi‐omic single cell analysis resolves novel stromal cell populations in healthy and diseased human tendon. Sci Rep. 2020;10(1):13939. 32883960 10.1038/s41598-020-70786-5 PMC7471282

[39] De Micheli AJ , Swanson JB , Disser NP , Martinez LM , Walker NR , Oliver DJ , et al. Single‐cell transcriptomic analysis identifies extensive heterogeneity in the cellular composition of mouse achilles tendons. Am J Physiol Cell Physiol. 2020;319(5):C885–C894. 32877217 10.1152/ajpcell.00372.2020 PMC7701267

[40] Lavagnino M , Madison J , Williams MR , Arnoczky SP . The equine forelimb suspensory ligament exhibits a heterogeneous strain pattern under tensile load. Vet Comp Orthop Traumatol. 2015;28(5):306–311. 26219950 10.3415/VCOT-15-01-0018

[41] Riemersma DJ , Van Den Bogert AJ , Jansen MO , Schamhardt HC . Tendon strain in the forelimbs as a function of gait and ground characteristics and in vitro limb loading in ponies. Equine Vet J. 1996;28(2):133–138. 8706645 10.1111/j.2042-3306.1996.tb01605.x

[42] Riemersma DJ , Van Den Bogert AJ , Jansen MO , Schamhardt HC . Influence of shoeing on ground reaction forces and tendon strains in the forelimbs of ponies. Equine Vet J. 1996;28(2):126–132. 8706644 10.1111/j.2042-3306.1996.tb01604.x

[43] Jansen MO , Schamhardt HC , Van Den Bogert AJ , Hartman W . Mechanical properties of the tendinous equine interosseus muscle are affected by in vivo transducer implantation. J Biomech. 1998;31(5):485–490. 9727347 10.1016/s0021-9290(98)00023-2

[44] Bardin AL , Tang L , Panizzi L , Rogers CW , Colborne GR . Development of an anybody musculoskeletal model of the Thoroughbred forelimb. J Equine Vet Sci. 2021;103:103666. 34281648 10.1016/j.jevs.2021.103666

[45] Takahashi T , Mukai K , Ohmura H , Aida H , Hiraga A . In vivo measurements of flexor tendon and suspensory ligament forces during trotting using the Thoroughbred forelimb model. J Equine Sci. 2014;25(1):15–22. 24834009 10.1294/jes.25.15 PMC4019201

[46] Meershoek LS , Lanovaz JL . Sensitivity analysis and application to trotting of a noninvasive method to calculate flexor tendon forces in the equine forelimb. Am J Vet Res. 2001;62(10):1594–1598. 11592325 10.2460/ajvr.2001.62.1594

[47] Walker VA , Walters JM , Griffith L , Murray RC . The effect of collection and extension on tarsal flexion and fetlock extension at trot. Equine Vet J. 2013;45(2):245–248. 22943288 10.1111/j.2042-3306.2012.00617.x

[48] Bardin AL , Taylor NC , Colborne GR . Response of the Thoroughbred forelimb to perturbations caused by a change in ground surface. J Equine Vet Sci. 2022;112:103897. 35150852 10.1016/j.jevs.2022.103897

[49] Firth EC , Rogers CW , Anderson BH . Musculoskeletal responses of 2‐year‐old Thoroughbred horses to early training. 4. Morphometric, microscopic and biomechanical properties of the digital tendons of the forelimb. N Z Vet J. 2004;52(5):285–292. 15768125 10.1080/00480169.2004.36441

[50] Birch HL , Mclaughlin L , Smith RK , Goodship AE . Treadmill exercise‐induced tendon hypertrophy: assessment of tendons with different mechanical functions. Equine Vet J. 1999;31(S30):222–226. 10.1111/j.2042-3306.1999.tb05222.x 10659256

[51] Johnston AS , Riggs CM , Cogger N , Benschop J , Rogers CW , Rosanowski SM . Using time‐series analysis techniques to enhance the understanding of musculoskeletal injury in Thoroughbred racehorses. Equine Vet J. 2020;52(5):699–708. 31811658 10.1111/evj.13220

[52] Welsh CE , Lewis TW , Blott SC , Mellor DJ , Lam KH , Stewart BD , et al. Preliminary genetic analyses of important musculoskeletal conditions of Thoroughbred racehorses in Hong Kong. Vet J. 2013;198(3):611–615. 23746478 10.1016/j.tvjl.2013.05.002 PMC3899015

[53] Kasashima Y , Takahashi T , Smith RK , Goodship AE , Kuwano A , Ueno T , et al. Prevalence of superficial digital flexor tendonitis and suspensory desmitis in Japanese Thoroughbred flat racehorses in 1999. Equine Vet J. 2004;36(4):346–350. 15163043 10.2746/0425164044890580

[54] Plevin S , Mclellan J . The effect of insertional suspensory branch desmitis on racing performance in juvenile Thoroughbred racehorses. Equine Vet J. 2014;46(4):451–457. 23937263 10.1111/evj.12161

[55] Hill AE , Blea JA , Arthur RM , Mcilwraith CW . Non‐fatal injury occurrence in southern California Thoroughbred racehorses 2009–2010. Vet J. 2015;205(1):98–100. 26026349 10.1016/j.tvjl.2015.04.001

[56] Ely ER , Avella CS , Price JS , Smith RK , Wood JL , Verheyen KL . Descriptive epidemiology of fracture, tendon and suspensory ligament injuries in national hunt racehorses in training. Equine Vet J. 2009;41(4):372–378. 19562899 10.2746/042516409x371224

[57] Hill AE , Stover SM , Gardner IA , Kane AJ , Whitcomb MB , Emerson AG . Risk factors for and outcomes of noncatastrophic suspensory apparatus injury in Thoroughbred racehorses. J Am Vet Med Assoc. 2001;218(7):1136–1144. 11318366 10.2460/javma.2001.218.1136

[58] Cohen ND , Mundy GD , Peloso JG , Carey VJ , Amend NK . Results of physical inspection before races and race‐related characteristics and their association with musculoskeletal injuries in Thoroughbreds during races. J Am Vet Med Assoc. 1999;215(5):654–661. 10476712

[59] Cohen ND , Peloso JG , Mundy GD , Fisher M , Holland RE , Little TV , et al. Racing‐related factors and results of prerace physical inspection and their association with musculoskeletal injuries incurred in Thoroughbreds during races. J Am Vet Med Assoc. 1997;211(4):454–463. 9267508

[60] Crawford KL , Finnane A , Greer RM , Phillips CJC , Woldeyohannes SM , Perkins NR , et al. Appraising the welfare of Thoroughbred racehorses in training in Queensland, Australia: the incidence and type of musculoskeletal injuries vary between two‐year‐old and older Thoroughbred racehorses. Animals. 2020;10(11):2046. 33167429 10.3390/ani10112046 PMC7694396

[61] Williams RB , Harkins LS , Wood JLN . Racehorse injuries, clinical problems and fatalities recorded on British racecourses from flat racing and national hunt racing during 1996, 1997 and 1998. Equine Vet J. 2001;33(5):478–486. 11558743 10.2746/042516401776254808

[62] Gibson KT , Burbidge HM , Pfeiffer DU . Superficial digital flexor tendonitis in Thoroughbred racehorses: outcome following non‐surgical treatment and superior check desmotomy. Aust Vet J. 1997;75:631–635. 9325536 10.1111/j.1751-0813.1997.tb15356.x

[63] Bertuglia A , Bullone M , Rossotto F , Gasparini M . Epidemiology of musculoskeletal injuries in a population of harness standardbred racehorses in training. BMC Vet Res. 2014;10(1):11. 24410888 10.1186/1746-6148-10-11 PMC3922780

[64] Kalisiak O . Parameters influencing prevalence and outcome of tendonitis in Thoroughbred and Arabian racehorses. Pol J Vet Sci. 2012;15(1):111–118. 22708365 10.2478/v10181-011-0121-9

[65] Dabareiner RM , Cohen ND , Carter GK , Nunn S , Moyer W . Musculoskeletal problems associated with lameness and poor performance among horses used for barrel racing: 118 cases (2000–2003). J Am Vet Med Assoc. 2005;227(10):1646–1650. 16313045 10.2460/javma.2005.227.1646

[66] Murray RC , Dyson SJ , Tranquille C , Adams V . Association of type of sport and performance level with anatomical site of orthopaedic injury diagnosis. Equine Vet J. 2006;38(S36):411–416. 10.1111/j.2042-3306.2006.tb05578.x 17402457

[67] Singer ER , Barnes J , Saxby F , Murray JK . Injuries in the event horse: training versus competition. Vet J. 2008;175(1):76–81. 17204438 10.1016/j.tvjl.2006.11.009

[68] Murray RC , Tranquille CA , Walker VA , Milmine RC , Bak L , Tacey JB , et al. Magnetic resonance imaging findings in the proximal metacarpal region of 359 horses and proximal metatarsal region of 64 horses acquired under standing sedation. J Equine Vet Sci. 2020;94:103268. 33077090 10.1016/j.jevs.2020.103268

[69] Van Den Belt AJ , Dik KJ , Barneveld A . Ultrasonographic evaluation and long‐term follow‐up of flexor tendonitis/desmitis in the metacarpal/metatarsal region in Dutch warmblood horses and standardbred racehorses. Vet Q. 1994;16(Suppl 2):S76–S80. 7801507

[70] Woodie JB , Ruggles AJ , Bertone AL , Hardy J , Schneider RK . Apical fracture of the proximal sesamoid bone in standardbred horses: 43 cases (1990–1996). J Am Vet Med Assoc. 1999;214(11):1653–1656. 10363098

[71] Auth AK , Hinnigan GJ , Smith MA , Owen KR . Low‐field magnetic resonance imaging findings of the fetlock region of nonracehorses. J Equine Vet Sci. 2024;132:104938. 37832699 10.1016/j.jevs.2023.104938

[72] Dyson S , Pinilla MJ , Bolas N , Murray R . Proximal suspensory desmopathy in hindlimbs: magnetic resonance imaging, gross post‐mortem and histological study. Equine Vet J. 2018;50(2):159–165. 28857286 10.1111/evj.12756

[73] Dyson S , Murray R , Pinilla M‐J . Proximal suspensory desmopathy in hindlimbs: a correlative clinical, ultrasonographic, gross post mortem and histological study. Equine Vet J. 2017;49(1):65–72. 26713512 10.1111/evj.12563

[74] O'Brien EJO , Smith RKW . Mineralization can be an incidental ultrasonographic finding in equine tendons and ligaments. Vet Radiol Ultrasound. 2018;59(5):613–623. 29776006 10.1111/vru.12628

[75] Bowser JE , Elder SH , Pasquali M , Grady JG , Rashmir‐Raven AM , Wills R , et al. Tensile properties in collagen‐rich tissues of quarter horses with hereditary equine regional dermal asthenia (herda). Equine Vet J. 2014;46(2):216–222. 23738970 10.1111/evj.12110

[76] Mero JL , Scarlett JM . Diagnostic criteria for degenerative suspensory ligament desmitis in Peruvian Paso horses. J Equine Vet Sci. 2005;25(5):224–228.

[77] Momen M , Brauer K , Patterson MM , Sample SJ , Binversie EE , Davis BW , et al. Genetic architecture and polygenic risk score prediction of degenerative suspensory ligament desmitis (DSLD) in the Peruvian horse. Front Genet. 2023;14:1201628. 37645058 10.3389/fgene.2023.1201628 PMC10460910

[78] Xie L , Spencer ND , Beadle RE , Gaschen L , Buchert MR , Lopez MJ . Effects of athletic conditioning on horses with degenerative suspensory ligament desmitis: a preliminary report. Vet J. 2011;189(1):49–57. 20655251 10.1016/j.tvjl.2010.06.010

[79] Halper J , Kim B , Khan A , Yoon JH , Mueller POE . Degenerative suspensory ligament desmitis as a systemic disorder characterized by proteoglycan accumulation. BMC Vet Res. 2006;2(1):12. 16611357 10.1186/1746-6148-2-12 PMC1459153

[80] Kim B , Yoon JH , Zhang J , Eric Mueller PO , Halper J . Glycan profiling of a defect in decorin glycosylation in equine systemic proteoglycan accumulation, a potential model of progeroid form of Ehlers–Danlos syndrome. Arch Biochem Biophys. 2010;501(2):221–231. 20599673 10.1016/j.abb.2010.06.017

[81] Plaas A , Sandy JD , Liu H , Diaz MA , Schenkman D , Magnus RP , et al. Biochemical identification and immunolocalizaton of aggrecan, ADAMTS5 and inter‐alpha‐trypsin‐inhibitor in equine degenerative suspensory ligament desmitis. J Orthop Res. 2011;29(6):900–906. 21246622 10.1002/jor.21332

[82] Luo W , Sandy J , Trella K , Gorski D , Gao S , Li J , et al. Degenerative suspensory ligament desmitis (DSLD) in Peruvian Paso horses is characterized by altered expression of TGFΒ signaling components in adipose‐derived stromal fibroblasts. PLoS One. 2016;11(11):e0167069. 27902739 10.1371/journal.pone.0167069 PMC5130251

[83] Young M , Moshood O , Zhang J , Sarbacher CA , Mueller POE , Halper J . Does bmp2 play a role in the pathogenesis of equine degenerative suspensory ligament desmitis? BMC Res Notes. 2018;11(1):672. 30227887 10.1186/s13104-018-3776-9 PMC6145121

[84] Haythorn A , Young M , Stanton J , Zhang J , Mueller POE , Halper J . Differential gene expression in skin RNA of horses affected with degenerative suspensory ligament desmitis. J Orthop Surg Res. 2020;15(1):460. 33028365 10.1186/s13018-020-01994-y PMC7541307

[85] Momen M , Brounts SH , Binversie EE , Sample SJ , Rosa GJM , Davis BW , et al. Selection signature analyses and genome‐wide association reveal genomic hotspot regions that reflect differences between breeds of horse with contrasting risk of degenerative suspensory ligament desmitis. G3 Genes Genom Genet. 2022;12(10):jkac179. 10.1093/g3journal/jkac179 PMC9526059 35866615

[86] Hofberger S , Gauff F , Licka T . Suspensory ligament degeneration associated with pituitary pars intermedia dysfunction in horses. Vet J. 2015;203(3):348–350. 25641552 10.1016/j.tvjl.2014.12.037

[87] Hofberger SC , Gauff F , Thaller D , Morgan R , Keen JA , Licka TF . Assessment of tissue‐specific cortisol activity with regard to degeneration of the suspensory ligaments in horses with pituitary pars intermedia dysfunction. Am J Vet Res. 2018;79(2):199–210. 29359976 10.2460/ajvr.79.2.199

[88] Manfredi JM , Jacob S , Norton E . A one‐health lens offers new perspectives on the importance of endocrine disorders in the equine athlete. J Am Vet Med Assoc. 2023;261(2):153–164. 36595370 10.2460/javma.22.11.0485

[89] Le Jeune SS , Macdonald MH , Stover SM , Taylor KT , Gerdes M . Biomechanical investigation of the association between suspensory ligament injury and lateral condylar fracture in Thoroughbred racehorses. Vet Surg. 2003;32(6):585–597. 14648539 10.1111/j.1532-950x.2003.00585.x

[90] Hill AE , Gardner IA , Carpenter TE , Lee CM , Hitchens PL , Stover SM . Prevalence, location and symmetry of noncatastrophic ligamentous suspensory apparatus lesions in California Thoroughbred racehorses, and association of these lesions with catastrophic injuries. Equine Vet J. 2016;48(1):27–32. 25290093 10.1111/evj.12367

[91] Spargo KE , Rubio‐Martinez LM , Wheeler DP , Fletcher L , Carstens A . Catastrophic musculoskeletal injuries in Thoroughbred racehorses on racetracks in Gauteng, South Africa. J S Afr Vet Assoc. 2019;90:e1–e5. 10.4102/jsava.v90i0.1640 PMC7081728 30843400

[92] Verschooten F , Gasthuys F , De Moor A . Distal splint bone fractures in the horse: an experimental and clinical study. Equine Vet J. 1984;16(6):532–536. 6519044 10.1111/j.2042-3306.1984.tb02011.x

[93] McLellan J , Plevin S . Do radiographic signs of sesamoiditis in yearling Thoroughbreds predispose the development of suspensory ligament branch injury? Equine Vet J. 2014;46(4):446–450. 23909567 10.1111/evj.12154

[94] Plevin S , McLellan J , O'Keeffe T . Association between sesamoiditis, subclinical ultrasonographic suspensory ligament branch change and subsequent clinical injury in yearling Thoroughbreds. Equine Vet J. 2016;48(5):543–547. 26282863 10.1111/evj.12497

[95] Bertoni L , Forresu D , Coudry V , Audigie F , Denoix J‐M . Exostoses on the palmar or plantar aspect of the diaphysis of the third metacarpal or metatarsal bone in horses: 16 cases (2001–2010). J Am Vet Med Assoc. 2012;240(6):740–747. 22380813 10.2460/javma.240.6.740

[96] Fairburn A , Dyson S , Murray R . Clinical significance of osseous spurs on the dorsoproximal aspect of the third metatarsal bone. Equine Vet J. 2010;42(7):591–599. 20840574 10.1111/j.2042-3306.2010.00097.x

[97] Dyson S . Proximal suspensory desmitis: clinical, ultrasonographic and radiographic features. Equine Vet J. 1991;23(1):25–31. 2015804 10.1111/j.2042-3306.1991.tb02708.x

[98] Ford TS , Ross MW , Orsini PG . A comparison of methods for proximal palmar metacarpal analgesia in horses. Vet Surg. 1989;18(2):146–150. 2728336 10.1111/j.1532-950x.1989.tb01059.x

[99] Gayle J , Redding R . Comparison of diagnostic anaesthetic techniques of the proximal plantar metatarsus in the horse. Equine Vet Educ. 2007;19:222–224.

[100] Dyson SJ , Romero JM . An investigation of injection techniques for local analgesia of the equine distal tarsus and proximal metatarsus. Equine Vet J. 1993;25(1):30–35. 8422881 10.1111/j.2042-3306.1993.tb02897.x

[101] Labens R , Schramme MC , Robertson ID , Thrall DE , Redding WR . Clinical, magnetic resonance, and sonographic imaging findings in horses with proximal plantar metatarsal pain. Vet Radiol Ultrasound. 2010;51(1):11–18. 20166387 10.1111/j.1740-8261.2009.01614.x

[102] Hughes TK , Eliashar E , Smith RK . In vitro evaluation of a single injection technique for diagnostic analgesia of the proximal suspensory ligament of the equine pelvic limb. Vet Surg. 2007;36(8):760–764. 18067616 10.1111/j.1532-950X.2007.00333.x

[103] Contino EK , King MR , Valdés‐Martínez A , Mcilwraith CW . In vivo diffusion characteristics following perineural injection of the deep branch of the lateral plantar nerve with mepivacaine or iohexol in horses. Equine Vet J. 2015;47(2):230–234. 24612216 10.1111/evj.12261

[104] Gerdes C , Morgan R , Terry R , Foote A , Smith R . Computed tomographic arthrography, gross anatomy and histology demonstrate a communication between synovial invaginations in the proximal aspect of the third interosseous muscle and the carpometacarpal joint in horses. Front Vet Sci. 2022;9:958598. 36118348 10.3389/fvets.2022.958598 PMC9478614

[105] Gaschen L , Burba DJ . Musculoskeletal injury in Thoroughbred racehorses: correlation of findings using multiple imaging modalities. Vet Clin North Am Equine Pract. 2012;28(3):539–561. 23177131 10.1016/j.cveq.2012.09.005

[106] Werpy N , Chapman K , Griffith L . Non‐weight bearing ultrasonographic examination allows the diagnosis of longitudinal fiber disruption (split) in equine suspensory ligament branches not visible on weight bearing examination. Vet Radiol Ultrasound. 2021;62(1):84–97. 33089588 10.1111/vru.12910

[107] Dyson S . Proximal suspensory desmitis in the hindlimb: 42 cases. Br Vet J. 1994;150(3):279–291. 8044668 10.1016/S0007-1935(05)80008-9

[108] Minshall GJ , Wright IM . Arthroscopic diagnosis and treatment of intra‐articular insertional injuries of the suspensory ligament branches in 18 horses. Equine Vet J. 2006;38(1):10–14. 16411580 10.2746/042516406775374243

[109] Dyson SJ , Arthur RM , Palmer SE , Richardson D . Suspensory ligament desmitis. Vet Clin North Am Equine Pract. 1995;11(2):177–215. 7584734 10.1016/s0749-0739(17)30319-x

[110] Ramzan PHL , Palmer L , Dallas RS , Shepherd MC . Subclinical ultrasonographic abnormalities of the suspensory ligament branch of the athletic horse: a survey of 60 Thoroughbred racehorses. Equine Vet J. 2013;45(2):159–163. 22607323 10.1111/j.2042-3306.2012.00588.x

[111] Fairburn AJ , Busschers E , Barr ARS . Subclinical ultrasonographic abnormalities of the suspensory ligament branches in national hunt racehorses. Equine Vet J. 2017;49(4):475–479. 27662244 10.1111/evj.12639

[112] Read RM , Boys‐Smith S , Bathe AP . Subclinical ultrasonographic abnormalities of the suspensory ligament branches are common in elite showjumping warmblood horses. Front Vet Sci. 2020;7:117. 32258068 10.3389/fvets.2020.00117 PMC7092662

[113] Rabba S , Petrucci V , Petrizzi L , Giommi DW , Busoni V . B‐mode ultrasonographic abnormalities and power Doppler signal in suspensory ligament branches of nonlame working quarter horses. J Equine Vet Sci. 2020;94:103254. 33077065 10.1016/j.jevs.2020.103254

[114] Zauscher JM , Estrada R , Edinger J , Lischer CJ . The proximal aspect of the suspensory ligament in the horse: how precise are ultrasonographic measurements? Equine Vet J. 2013;45(2):164–169. 22784195 10.1111/j.2042-3306.2012.00597.x

[115] Werpy NM , Denoix JM , Mcilwraith CW , Frisbie DD . Comparison between standard ultrasonography, angle contrast ultrasonography, and magnetic resonance imaging characteristics of the normal equine proximal suspensory ligament. Vet Radiol Ultrasound. 2013;54(5):536–547. 23718137 10.1111/vru.12051

[116] Sullivan HM , Barrett MF , Zhou T , Kawcak CE . Ultrasonographic evaluation of the suspensory ligament in quarter horses used for cutting. J Equine Vet Sci. 2022;119:104139. 36252794 10.1016/j.jevs.2022.104139

[117] Rabba S , Grulke S , Verwilghen D , Evrard L , Busoni V . B‐mode and power Doppler ultrasonography of the equine suspensory ligament branches: a descriptive study on 13 horses. Vet Radiol Ultrasound. 2018;59(4):453–460. 29498123 10.1111/vru.12610

[118] Lustgarten M , Redding WR , Labens R , Morgan M , Davis W , Seiler GS . Elastographic characteristics of the metacarpal tendons in horses without clinical evidence of tendon injury. Vet Radiol Ultrasound. 2014;55(1):92–101. 24103015 10.1111/vru.12104

[119] Lustgarten M , Redding WR , Labens R , Davis W , Daniel TM , Griffith E , et al. Elastographic evaluation of naturally occurring tendon and ligament injuries of the equine distal limb. Vet Radiol Ultrasound. 2015;56(6):670–679. 26304065 10.1111/vru.12284

[120] Kleiter M , Kneissl S , Stanek C , Mayrhofer E , Baulain U , Deegen E . Evaluation of magnetic resonance imaging techniques in the equine digit. Vet Radiol Ultrasound. 1999;40(1):15–22. 10023990 10.1111/j.1740-8261.1999.tb01833.x

[121] Nagy A , Dyson S . Magnetic resonance imaging and histological findings in the proximal aspect of the suspensory ligament of forelimbs in nonlame horses. Equine Vet J. 2012;44(1):43–50. 21649714 10.1111/j.2042-3306.2011.00365.x

[122] Zubrod CJ , Barrett MF . Magnetic resonance imaging of tendon and ligament injuries. Clin Tech Equine Pract. 2007;6(3):217–229.

[123] Brokken MT , Schneider RK , Sampson SN , Tucker RL , Gavin PR , Ho CP . Magnetic resonance imaging features of proximal metacarpal and metatarsal injuries in the horse. Vet Radiol Ultrasound. 2007;48(6):507–517. 18018721 10.1111/j.1740-8261.2007.00288.x

[124] Nagy A , Dyson S . Magnetic resonance imaging findings in the carpus and proximal metacarpal region of 50 lame horses. Equine Vet J. 2012;44(2):163–168. 21895751 10.1111/j.2042-3306.2011.00422.x

[125] Nagy A , Dyson S . Magnetic resonance anatomy of the proximal metacarpal region of the horse described from images acquired from low‐ and high‐field magnets. Vet Radiol Ultrasound. 2009;50(6):595–605. 19999342 10.1111/j.1740-8261.2009.01589.x

[126] Gonzalez LM , Schramme MC , Robertson ID , Thrall DE , Redding RW . MRI features of metacarpo(tarso)phalangeal region lameness in 40 horses. Vet Radiol Ultrasound. 2010;51(4):404–414. 20806872 10.1111/j.1740-8261.2010.01676.x

[127] King JN , Zubrod CJ , Schneider RK , Sampson SN , Roberts G . MRI findings in 232 horses with lameness localized to the metacarpo(tarso)phalangeal region and without a radiographic diagnosis. Vet Radiol Ultrasound. 2013;54(1):36–47. 23020207 10.1111/j.1740-8261.2012.01983.x

[128] Labens R , Schramme MC , Murray RC , Bolas N . Standing low‐field MRI of the equine proximal metacarpal/metatarsal region is considered useful for diagnosing primary bone pathology and makes a positive contribution to case management: a prospective survey study. Vet Radiol Ultrasound. 2020;61(2):197–205. 31800146 10.1111/vru.12824

[129] Barrett MF , Manchon PT , Hersman J , Kawcak CE . Magnetic resonance imaging findings of the proximal metacarpus in quarter horses used for cutting: retrospective analysis of 32 horses 2009–2012. Equine Vet J. 2018;50(2):172–178. 28833365 10.1111/evj.12746

[130] Zubrod CJ , Schneider RK , Tucker RL . Use of magnetic resonance imaging identify suspensory desmitis and adhesions between exostoses of the second metacarpal bone and the suspensory ligament in four horses. J Am Vet Med Assoc. 2004;224(11):1815–1820, 1789. 15198268 10.2460/javma.2004.224.1815

[131] Barrett MF , Selberg KT , Johnson SA , Hersman J , Frisbie DD . High field magnetic resonance imaging contributes to diagnosis of equine distal tarsus and proximal metatarsus lesions: 103 horses. Vet Radiol Ultrasound. 2018;59(5):587–596. 30027637 10.1111/vru.12659

[132] Hinkle FE , Selberg KT , Frisbie DD , Barrett MF . Radiographic changes of the proximal third metatarsal bone do not predict presence or severity of proximal suspensory desmopathy in a predominately quarter horse population. Equine Vet J. 2023;55(1):24–32. 35092318 10.1111/evj.13562

[133] Van Veggel E , Selberg K , Van Der Velde‐Hoogelander B , Bolas N , Vanderperren K , Bergman HJ . Magnetic resonance imaging findings of the proximal metacarpal region in warmblood horses: 36 lame and 26 control limbs (2015–2021). Front Vet Sci. 2021;8:714423. 34458356 10.3389/fvets.2021.714423 PMC8388851

[134] Launois MT , Vandeweerd J‐MEF , RaR P , Brogniez L , Desbrosse FG , Clegg PD . Use of computed tomography to diagnose new bone formation associated with desmitis of the proximal aspect of the suspensory ligament in third metacarpal or third metatarsal bones of three horses. J Am Vet Med Assoc. 2009;234(4):514–518. 19222363 10.2460/javma.234.4.514

[135] Lin S‐T , Peter VG , Schiavo S , Pokora R , Patrick H , Bolas N , et al. Identification of heterotopic mineralization and adjacent pathology in the equine fetlock region by low‐field magnetic resonance imaging, cone‐beam and fan‐beam computed tomography. J Equine Vet Sci. 2023;126:104252. 36796738 10.1016/j.jevs.2023.104252

[136] Müller EMT , Vanderperren K , Merle R , Rheinfeld S , Leelamankong P , Lischer CJ , et al. Findings consistent with equine proximal suspensory desmitis can be reliably detected using computed tomography and differ between affected horses and controls. Vet Radiol Ultrasound. 2023;64(6):E64–E96. 37605336 10.1111/vru.13292

[137] Edwards RB 3rd , Ducharme NG , Fubini SL , Yeager AE , Kallfelz FA . Scintigraphy for diagnosis of avulsions of the origin of the suspensory ligament in horses: 51 cases (1980–1993). J Am Vet Med Assoc. 1995;207(5):608–611. 7649776

[138] Williams J , Miyabayashi T , Ruggles A , Yamamoto J , Takiguchi M . Scintigraphic and ultrasonographic diagnosis of soft tissue injury in a Thoroughbred horse. J Vet Med Sci. 1994;56(1):169–172. 8204749 10.1292/jvms.56.169

[139] Dyson SJ , Weekes JS , Murray RC . Scintigraphic evaluation of the proximal metacarpal and metatarsal regions of horses with proximal suspensory desmitis. Vet Radiol Ultrasound. 2007;48(1):78–85. 17236365 10.1111/j.1740-8261.2007.00208.x

[140] Oomen AM , Oosterlinck M , Pille F , Sonneveld DC , Gasthuys F , Back W . Use of a pressure plate to analyse the toe–heel load redistribution underneath a normal shoe and a shoe with a wide toe in sound warmblood horses at the walk and trot. Res Vet Sci. 2012;93(2):1026–1031. 22342126 10.1016/j.rvsc.2012.01.010

[141] Lawson SE , Chateau H , Pourcelot P , Denoix JM , Crevier‐Denoix N . Effect of toe and heel elevation on calculated tendon strains in the horse and the influence of the proximal interphalangeal joint. J Anat. 2007;210(5):583–591. 17451533 10.1111/j.1469-7580.2007.00714.x PMC2375746

[142] Kadic DTN , Minshall GJ , Wright IM . Surgical management of marginal tears/avulsions of the suspensory ligament branches in 29 Thoroughbred racehorses. Equine Vet J. 2019;51(3):310–315. 30206960 10.1111/evj.13020

[143] Hewes CA , White NA . Outcome of desmoplasty and fasciotomy for desmitis involving the origin of the suspensory ligament in horses: 27 cases (1995–2004). J Am Vet Med Assoc. 2006;229(3):407–412. 16881834 10.2460/javma.229.3.407

[144] Brokken MT , Schneider RK , Roberts GD , Holmes SP , Gavin PR , Sampson SN , et al. Evaluation of a new surgical treatment for equine hind limb proximal suspensory desmitis. Vet Surg. 2016;45(7):868–878. 27545972 10.1111/vsu.12527

[145] Tóth F , Schumacher J , Schramme M , Holder T , Adair HS , Donnell RL . Compressive damage to the deep branch of the lateral plantar nerve associated with lameness caused by proximal suspensory desmitis. Vet Surg. 2008;37(4):328–335. 18564256 10.1111/j.1532-950X.2008.00385.x

[146] Guasco PG , Kelly G , Schumacher J , Henry RW . Excision of the deep branch of the lateral palmar nerve of horses to resolve lameness caused by proximal suspensory desmitis. Vet Surg. 2013;42(3):296–301. 23241073 10.1111/j.1532-950X.2012.01073.x

[147] Scharf A , De Solis CN , Sampson SN , Glass K , Watts AE . Suspensory ligament size does not change after plantar fasciotomy and neurectomy of the deep branch of the lateral plantar nerve by ultrasonographic assessment. Vet Surg. 2022;51(2):259–269. 34970755 10.1111/vsu.13757 PMC9306907

[148] Tatarniuk DM , Hill JA , Modesto RB , Swor TM , Caston SS , Kersh KD . Outcome following neurectomy of the deep branch lateral plantar nerve and plantar fasciotomy for hindlimb proximal suspensory desmopathy in western performance horses: 21 cases. Vet Surg. 2021;50(2):273–282. 33331004 10.1111/vsu.13552

[149] Dyson S , Murray R . Management of hindlimb proximal suspensory desmopathy by neurectomy of the deep branch of the lateral plantar nerve and plantar fasciotomy: 155 horses (2003–2008). Equine Vet J. 2012;44(3):361–367. 21883416 10.1111/j.2042-3306.2011.00445.x

[150] José Antonio G , Jim S , Ramés SJ , Rohrbach BW , Alejandro Rodríguez M , Laura Romero R , et al. Denervating the pelvic suspensory ligaments of horses causes morphological and histological changes in the ligaments. Am J Vet Res. 2022;83(5):399–404. 35202000 10.2460/ajvr.21.09.0148

[151] Lopez‐Navarro G , Trigo‐Tavera FJ , Rodriguez‐Monterde A , Gutierrez‐Ospina G , Arechavaleta‐Velasco M , Schumacher J , et al. Histological changes in the proximal suspensory ligament after neurectomy of the deep branch of the lateral palmar nerve of horses with induced proximal suspensory desmitis. Vet J. 2017;227:46–48. 29031330 10.1016/j.tvjl.2017.08.008

[152] Pauwels FE , Schumacher J , Mayhew IG , Van Sickle DC . Neurectomy of the deep branch of the lateral plantar nerve can cause neurogenic atrophy of the muscle fibres in the proximal part of the suspensory ligament (M. Interosseous III). Equine Vet J. 2009;41(5):508–510. 19642414 10.2746/042516409x435629

[153] Crowe OM , Dyson SJ , Wright IM , Schramme MC , Smith RKW . Treatment of chronic or recurrent proximal suspensory desmitis using radial pressure wave therapy in the horse. Equine Vet J. 2004;36(4):313–316. 15163037 10.2746/0425164044890562

[154] Lischer CJ , Ringer SK , Schnewlin M , Imboden I , Fürst A , Stöckli M , et al. Treatment of chronic proximal suspensory desmitis in horses using focused electrohydraulic shockwave therapy. Schweiz Arch Tierheilkd. 2006;148(10):561–568. 17076464 10.1024/0036-7281.148.10.561

[155] Giunta K , Donnell JR , Donnell AD , Frisbie DD . Prospective randomized comparison of platelet rich plasma to extracorporeal shockwave therapy for treatment of proximal suspensory pain in western performance horses. Res Vet Sci. 2019;126:38–44. 31430578 10.1016/j.rvsc.2019.07.020

[156] Imboden I , Waldern NM , Wiestner T , Lischer CJ , Ueltschi G , Weishaupt MA . Short term analgesic effect of extracorporeal shock wave therapy in horses with proximal palmar metacarpal/plantar metatarsal pain. Vet J. 2009;179(1):50–59. 18069025 10.1016/j.tvjl.2007.09.020

[157] McClure SR , Vansickle D , Evans R , Reinertson EL , Moran L . The effects of extracorporeal shock‐wave therapy on the ultrasonographic and histologic appearance of collagenase‐induced equine forelimb suspensory ligament desmitis. Ultrasound Med Biol. 2004;30(4):461–467. 15121248 10.1016/j.ultrasmedbio.2003.12.005

[158] Caminoto EH , Alves AL , Amorim RL , Thomassian A , Hussni CA , Nicoletti JL . Ultrastructural and immunocytochemical evaluation of the effects of extracorporeal shock wave treatment in the hind limbs of horses with experimentally induced suspensory ligament desmitis. Am J Vet Res. 2005;66(5):892–896. 15934618 10.2460/ajvr.2005.66.892

[159] Bosch G , Lin YL , Van Schie HTM , Van De Lest CHA , Barneveld A , Van Weeren PR . Effect of extracorporeal shock wave therapy on the biochemical composition and metabolic activity of tenocytes in normal tendinous structures in ponies. Equine Vet J. 2007;39(3):226–231. 17520973 10.2746/042516407x180408

[160] Bosch G , De Mos M , Van Binsbergen R , Van Schie HTM , Van De Lest CHA , Van Weeren PR . The effect of focussed extracorporeal shock wave therapy on collagen matrix and gene expression in normal tendons and ligaments. Equine Vet J. 2009;41(4):335–341. 19562893 10.2746/042516409x370766

[161] Pluim M , Martens A , Vanderperren K , Sarrazin S , Koene M , Luciani A , et al. Short‐ and long term follow‐up of 150 sports horses diagnosed with tendinopathy or desmopathy by ultrasonographic examination and treated with high‐power laser therapy. Res Vet Sci. 2018;119:232–238. 30005398 10.1016/j.rvsc.2018.06.003

[162] Pluim M , Martens A , Vanderperren K , Van Weeren R , Oosterlinck M , Dewulf J , et al. High‐power laser therapy improves healing of the equine suspensory branch in a standardized lesion model. Front Vet Sci. 2020;7:600. 33102552 10.3389/fvets.2020.00600 PMC7494822

[163] Pluim M , Heier A , Plomp S , Boshuizen B , Gröne A , Van Weeren R , et al. Histological tissue healing following high‐power laser treatment in a model of suspensory ligament branch injury. Equine Vet J. 2022;54(6):1114–1122. 35008124 10.1111/evj.13556

[164] Carrozzo U , Toniato M , Harrison A . Assessment of noninvasive low‐frequency ultrasound as a means of treating injuries to suspensory ligaments in horses: a research paper. J Equine Vet Sci. 2019;80:80–89. 31443840 10.1016/j.jevs.2019.07.007

[165] Vlahos TP . Percutaneous ultrasonic debridement of equine tendinopathy and desmopathy: a report of 10 cases. Open Vet J. 2023;13(9):1141–1149. 37842115 10.5455/OVJ.2023.v13.i9.10 PMC10576587

[166] Argüelles D , Carmona JU , Climent F , Muñoz E , Prades M . Autologous platelet concentrates as a treatment for musculoskeletal lesions in five horses. Vet Rec. 2008;162(7):208–211. 18281627 10.1136/vr.162.7.208

[167] Castelijns G , Crawford A , Schaffer J , Ortolano GA , Beauregard T , Smith RK . Evaluation of a filter‐prepared platelet concentrate for the treatment of suspensory branch injuries in horses. Vet Comp Orthop Traumatol. 2011;24(5):363–369. 21887455 10.3415/VCOT-11-01-0001

[168] Waselau M , Sutter W , Genovese RL , Bertone AL . Intralesional injection of platelet‐rich plasma followed by controlled exercise for treatment of midbody suspensory ligament desmitis in standardbred racehorses. J Am Vet Med Assoc. 2008;232(10):1515–1520. 18479242 10.2460/javma.232.10.1515

[169] Garrett KS , Bramlage LR , Spike‐Pierce DL , Cohen ND . Injection of platelet‐ and leukocyte‐rich plasma at the junction of the proximal sesamoid bone and the suspensory ligament branch for treatment of yearling Thoroughbreds with proximal sesamoid bone inflammation and associated suspensory ligament branch desmitis. J Am Vet Med Assoc. 2013;243(1):120–125. 23786200 10.2460/javma.243.1.120

[170] Maleas G , Mageed M . Effectiveness of platelet‐rich plasma and bone marrow aspirate concentrate as treatments for chronic hindlimb proximal suspensory desmopathy. Front Vet Sci. 2021;8:678453. 34222402 10.3389/fvets.2021.678453 PMC8253571

[171] Hall M , Vasey J , Russell J , Russell T . Use of ultrasound‐guided autologous bone marrow transfer for treatment of suspensory ligament desmitis in 30 race horses (2003–2010). Aust Vet J. 2013;91(3):102–107. 23438462 10.1111/avj.12015

[172] Torricelli P , Fini M , Filardo G , Tschon M , Pischedda M , Pacorini A , et al. Regenerative medicine for the treatment of musculoskeletal overuse injuries in competition horses. Int Orthop. 2011;35(10):1569–1576. 21394594 10.1007/s00264-011-1237-3 PMC3174295

[173] Van Loon VJ , Scheffer CJ , Genn HJ , Hoogendoorn AC , Greve JW . Clinical follow‐up of horses treated with allogeneic equine mesenchymal stem cells derived from umbilical cord blood for different tendon and ligament disorders. Vet Q. 2014;34(2):92–97. 25072527 10.1080/01652176.2014.949390

[174] Hansen SH , Bramlage LR , Moore GE . Racing performance of Thoroughbred racehorses with suspensory ligament branch desmitis treated with mesenchymal stem cells (2010–2019). Equine Vet J. 2023;56(3):503–513. 37534804 10.1111/evj.13980

[175] Depuydt E , Chiers K , Van Hecke L , Saunders J , Martens A , Pille F , et al. Assessing the functional properties of tenogenic primed mesenchymal stem cells in ex vivo equine tendon and ligament explants: a preliminary study. Stem Cell Res. 2022;65:102963. 36395687 10.1016/j.scr.2022.102963

[176] Vandenberghe A , Broeckx SY , Beerts C , Seys B , Zimmerman M , Verweire I , et al. Tenogenically induced allogeneic mesenchymal stem cells for the treatment of proximal suspensory ligament desmitis in a horse. Front Vet Sci. 2015;2:49. 26664976 10.3389/fvets.2015.00049 PMC4672201

[177] Beerts C , Suls M , Broeckx SY , Seys B , Vandenberghe A , Declercq J , et al. Tenogenically induced allogeneic peripheral blood mesenchymal stem cells in allogeneic platelet‐rich plasma: 2‐year follow‐up after tendon or ligament treatment in horses. Front Vet Sci. 2017;4:158. 29018808 10.3389/fvets.2017.00158 PMC5622984

[178] Carlier S , Depuydt E , Suls M , Bocqué C , Thys J , Vandenberghe A , et al. Equine allogeneic tenogenic primed mesenchymal stem cells: a clinical field study in horses suffering from naturally occurring superficial digital flexor tendon and suspensory ligament injuries. Equine Vet J. 2023;56(5):924–935. 37847100 10.1111/evj.14008

[179] Kornicka‐Garbowska K , Pędziwiatr R , Woźniak P , Kucharczyk K , Marycz K . Microvesicles isolated from 5‐azacytidine‐and‐resveratrol‐treated mesenchymal stem cells for the treatment of suspensory ligament injury in horse—a case report. Stem Cell Res Ther. 2019;10(1):394. 31852535 10.1186/s13287-019-1469-5 PMC6921487

[180] Kovac M , Litvin YA , Aliev RO , Zakirova EY , Rutland CS , Kiyasov AP , et al. Gene therapy using plasmid DNA encoding VEGF164 and FGF2 genes: a novel treatment of naturally occurring tendinitis and desmitis in horses. Front Pharmacol. 2018;9:978. 30233367 10.3389/fphar.2018.00978 PMC6127648

[181] Kovac M , Litvin YA , Aliev RO , Zakirova EY , Rutland CS , Kiyasov AP , et al. Gene therapy using plasmid DNA encoding vascular endothelial growth factor 164 and fibroblast growth factor 2 genes for the treatment of horse tendinitis and desmitis: case reports. Front Vet Sci. 2017;4:168. 29067288 10.3389/fvets.2017.00168 PMC5641304

